# NTF4 plays a dual role in breast cancer in mammary tumorigenesis and metastatic progression

**DOI:** 10.7150/ijbs.79435

**Published:** 2023-01-01

**Authors:** Ran Sun, Jin He, Qin Xiang, Yixiao Feng, Yijia Gong, Yijiao Ning, Chaoqun Deng, Kexin Sun, Mingjun Zhang, Zhaobo Cheng, Xin Le, Qi Xiong, Fengsheng Dai, Yongzhong Wu, Tingxiu Xiang

**Affiliations:** 1 Department of Oncology, The First Affiliated Hospital of Chongqing Medical University, Chongqing 400016, China.; 2 Department of Oncology, Jiulongpo People's Hospital, Chongqing 400050, China.; 3 Department of Laboratory Medicine, Jiulongpo People's Hospital, Chongqing 400050, China.; 4 Chongqing Key Laboratory of Translational Research for Cancer Metastasis and Individualized Treatment, Chongqing University Cancer Hospital, Chongqing 400030, China.

**Keywords:** Breast cancer, NTF4, epithelial-mesenchymal transition, metastasis, apoptosis

## Abstract

Breast cancer metastasis can happen even when the primary tumor is relatively small. But the mechanism for such early metastasis is poorly understood. Herein, we report that neurotrophin 4 (NTF4) plays a dual role in breast cancer proliferation and metastasis. Clinical data showed high levels of NTF4, especially in the early stage, to be associated with poor clinical outcomes, supporting the notion that metastasis, rather than primary cancer, was the major determinant of breast cancer mortality for patients. NTF4 promoted epithelial-mesenchymal transition (EMT), cell motility, and invasiveness of breast cancer cells* in vitro* and *in vivo*. Interestingly, NTF4 inhibited cell proliferation while promoting cellular apoptosis *in vitro* and inhibited xenograft tumorigenicity *in vivo*. Mechanistically, NTF4 elicited its pro-metastatic effects by activating PRKDC/AKT and ANXA1/NF-κB pathways to stabilize SNAIL protein, therefore decreasing the level of E-cadherin. Conversely, NTF4 increased ANXA1 phosphorylation and sumoylation and the interaction with importin β, leading to nuclear import and retention of ANXA1, which in turn activates the caspase-3 apoptosis cascade. Our findings identified an unexpected dual role for NTF4 in breast cancer which contributes to early metastasis of the disease. Therefore, NTF4 may serve as a prognostic marker and a potential therapeutic target for breast cancer.

## Introduction

Malignant progression and metastasis of breast cancer are highly complex processes involving primary tumor growth and invasion followed by metastatic dissemination to distant organs 1-3. Clinical evidence suggests that distant metastasis rather than primary tumors accounts for the vast majority of breast cancer-related mortality 2, 3. To improve clinical outcomes, it is urgent to identify molecular determinants governing breast cancer metastatic progression and to explore novel therapeutic targets for anti-metastatic therapy. Notably, breast cancer is a highly clinical heterogeneous disease with distinct molecular subtypes and metastatic behaviors 2, 4. Based on the expression of estrogen receptor (ER), progesterone receptor (PR), and human epidermal growth receptor 2 (HER2), breast cancer is clinically divided into four major molecular subtypes: luminal A, Luminal B, HER2-positive, and triple-negative breast cancer (TNBC) 2, 4. Generally, luminal breast cancers tend to have a lower probability of metastatic spread and the better outcomes, while HER2-positive and TNBC subtypes have a higher propensity for metastatic progression and are associated with a poorer prognosis 2, 5.

Considerable evidence shows that aberrant activation of the oncogenic epithelial-mesenchymal transition (EMT) program results in invasion and metastasis of breast cancer 6. EMT is characterized by the loss of cancer cell epithelial characteristics and the acquisition of mesenchymal phenotypes with enhanced migratory and invasive properties 7. This phenotypic switch in cellular differentiation and behavior is mediated by key transcription factors including: SNAIL, TWIST, and ZEB. These transcription factors are finely regulated at the transcriptional, translational, and post-translational levels 8. The reprogramming of gene expression during EMT, as well as non-transcriptional modifications, are initiated and controlled by signaling pathways that respond to extracellular cues. Of these, activated AKT kinase acts as a “Master Regulator” that is necessary to metastasis 8. The AKT metastasis signaling network plays a central role in a variety of oncogenic processes including: cell growth, proliferation, apoptotic cell death, motility, EMT, angiogenesis and metastasis 8.

The neurotrophins constitute a family of structurally and functionally related polypeptides that includes: nerve growth factor (NGF), brain derived neurotrophic factor (BDNF), neurotrophin-3 (NTF3), and neurotrophin-4 (NTF4). Neurotrophins play crucial roles in the development and maintenance of the nervous system, where they stimulate neuronal cell survival, differentiation, and plasticity. Neurotrophins and their receptors participate in the tumorigenesis of multiple carcinomas 9-15 including breast cancer 16-19. NTF4 contributes to breast cancer cell survival by mediating cell resistance to apoptosis 20. However, no studies have systematically investigated a role for NTF4 in the tumorigenesis and metastatic progression of breast cancer.

Distant metastases, rather than primary cancer, is not only the major determinant of breast cancer mortality for patients, but also difficult to be identified at early stage. Therefore, it is urgent to be made breakthrough in early diagnosis and treatment. In this study, we reported that NTF4 was downregulated in primary breast tumors but upregulated in normal breast tissues and in metastatic tissues. Breast cancer patients with high levels of NTF4, especially in the early stage, have a poorer clinical prognosis. On the one hand, NTF4 elicited its pro-metastatic effects by activating PRKDC/AKT and ANXA1/NF-κB pathways to increase SNAIL stability, thereby decreasing E-cadherin. On the other hand, NTF4 exerted its anti-proliferative effect by increasing the phosphoserine and sumoylation levels of ANXA1 and the interactions between ANXA1 and importin β, which promotes ANXA1 nuclear translocation and activation of the caspase-3 apoptotic cascade. In other words, NTF4 can induce apoptosis of precancerous cells and inhibit the proliferation of cancer cells. However, when tumors progress, NTF4 promotes EMT and invasion and metastasis of tumor cells. These findings identify two unexpected separate functions for NTF4 that impact breast cancer progression and metastasis, and the upregulation of NTF4 in early breast cancer patients may serve as a predictive biomarker for progression or metastasis.

## Results

### NTF4 is downregulated in primary breast cancer, but upregulated with metastasis

**Figure 1:**
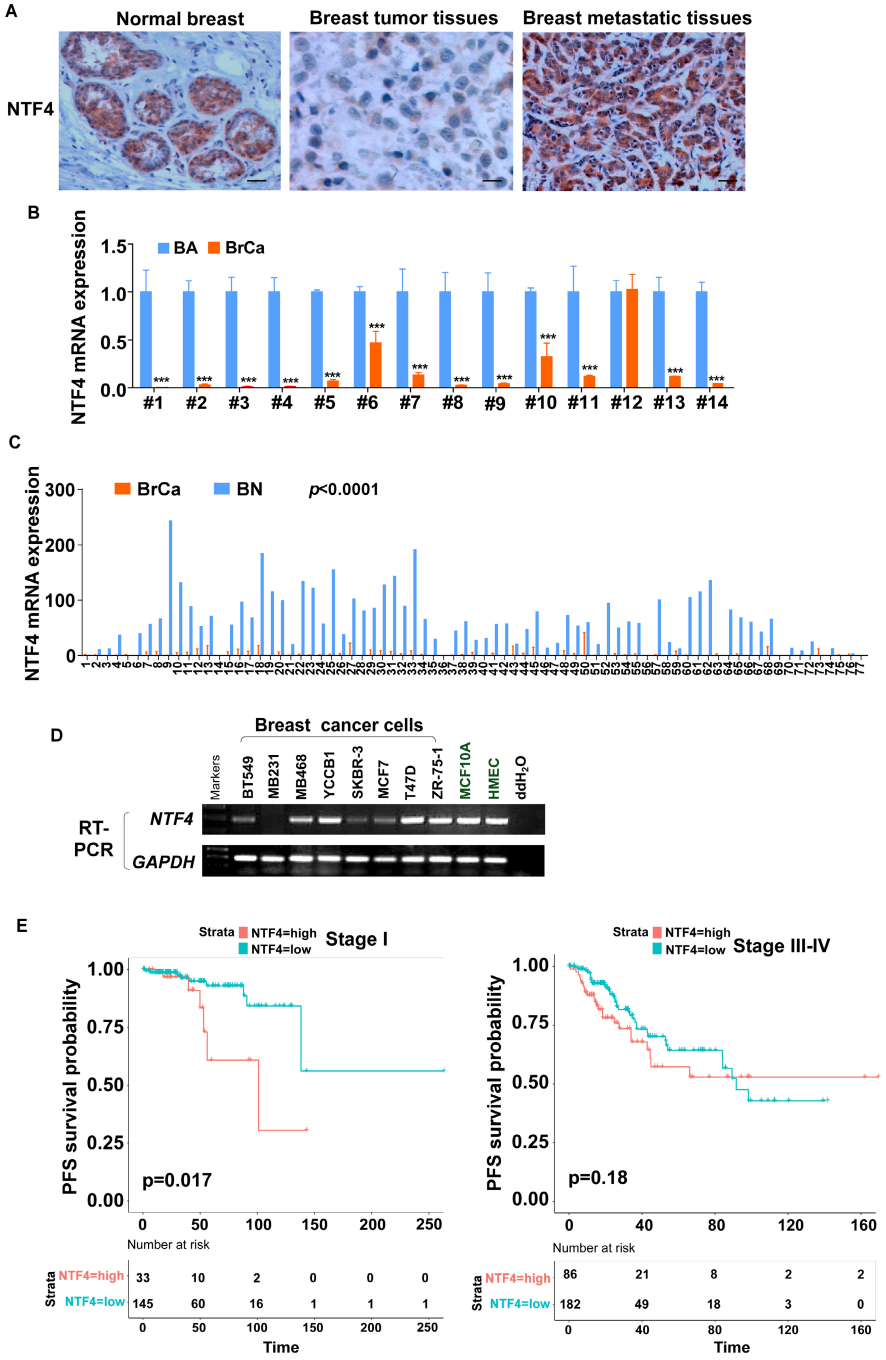
** NTF4 expression is associated with the prognosis of patients with breast cancer. (A)** IHC analysis was carried out by using breast cancer samples. Representative IHC images of NTF4 expression are shown. **(B)** qRT-PCR analysis of NTF4 mRNA expression in 14 paired primary breast cancer tissues (BrCa) and adjacent normal tissues (BA). **(C)** TCGA analysis of NTF4 mRNA expression in 77 paired primary breast cancer tissues (BrCa) and adjacent normal tissues (BN). **(D)** RT-PCR analysis of NTF4 expression in breast cancer cells and immortalized epithelial cells. **(E)** Kaplan-Meier curves of OS and PFS of breast cancer patients with high or low NTF4 expression. Data are presented as the mean±SD. ***p<0.001.

To investigate novel, potentially tumor related genes, paired breast cancers, normal tissues, and metastatic tissues were screened by RNA-seq. Expression of NTF4 mRNA was found to be remarkably downregulated in breast cancer tissues compared to normal breast tissues and adjacent normal tissues, but was upregulated in metastatic breast tissues 21 (**Fig. S1A**). Immunohistochemistry (IHC) staining was performed with 50 paired primary breast cancer tissues and adjacent normal tissues, as well as 15 metastatic lymph nodes. NTF4 was found to be localized primarily in the cytoplasm. Significantly decreased expression of NTF4 was observed in primary breast cancer tissue, with increased expression in metastatic lymph nodes (**Fig. 1A**), which was consistent with results of the RNA-Seq screening.

The expression levels of NTF4 in 14 matched pairs of human breast cancer and normal breast tissue were assessed by quantitative real-time polymerase chain reaction (qRT-PCR). Results showed that NTF4 mRNA was downregulated in 13 of 14 (92.86%) primary breast tumor tissues compared to their normal counterparts (**Fig. 1B**), which is consistent with results obtained by MethHC 2.0 22, 23 (**Fig. 1C**). We also determined the expression levels of NTF4 in two normal human mammary epithelial cell lines and eight well-characterized breast cancer cell lines. As shown in (**Fig. 1D**), normal mammary epithelial cell lines (MCF10A and HMEC), luminal-type breast cancer cell lines (T47D, ZR-75-1), and the metastatic cell line (MDA-MB-468) expressed relatively high levels of NTF4 compared to the other cell lines.

To assess the association between NTF4 expression and breast cancer patient survival, Kaplan-Meier curves with log-rank test were used to determine overall survival (OS), distant-metastasis-free survival (DMFS), and progression-free survival (PFS) of breast cancer patients derived in different stage from TCGA (The Cancer Genome Atlas, https://portal.gdc.cancer.gov/). The group with the highest levels of NTF4 tumor expression had poorer OS and DMFS compared to the group with the least NTF4 expression (**Fig. S1B**). Furthermore, subgroup survival analysis at stage I showed the group with high NTF4 expression to have poorer PFS (*p*<0.05), suggesting that the upregulation of NTF4 in early breast cancer patients may serve as a predictive biomarker for progression or metastasis (**Fig. 1E**). What's more, by using Kaplan-Meier Plotter dataset (https://kmplot.com/analysis/), we found that patients with high expression of NTF4 have poorer OS and recurrence-free survival (RFS) in Luminal A and and Basal (TNBC) breast cancer, and poorer RFS in HER2-positive breast cancer (*p*<0.05) (**Fig. S2**) 24. And we found that patients with high expression of NTF4 have poorer RFS in Grade 2 breast cancer, and poorer OS and RFS in Grade 3 breast cancer (*p*<0.05) (**Fig. S3**) 24. The area under the ROC curve suggested that NTF4 had accuracy for prediction of breast cancer (AUC=0.839, CI: 0.785-0.839) (**Fig. S1C**). Based on the above clinical data, breast cancer patients with high levels of NTF4 have a poorer clinical prognosis and that NTF4 may be a useful marker for predictive early breast cancer risk for metastasis.

### NTF4 suppresses breast cancer cell proliferation *in vitro* and *in vivo*

**Figure 2:**
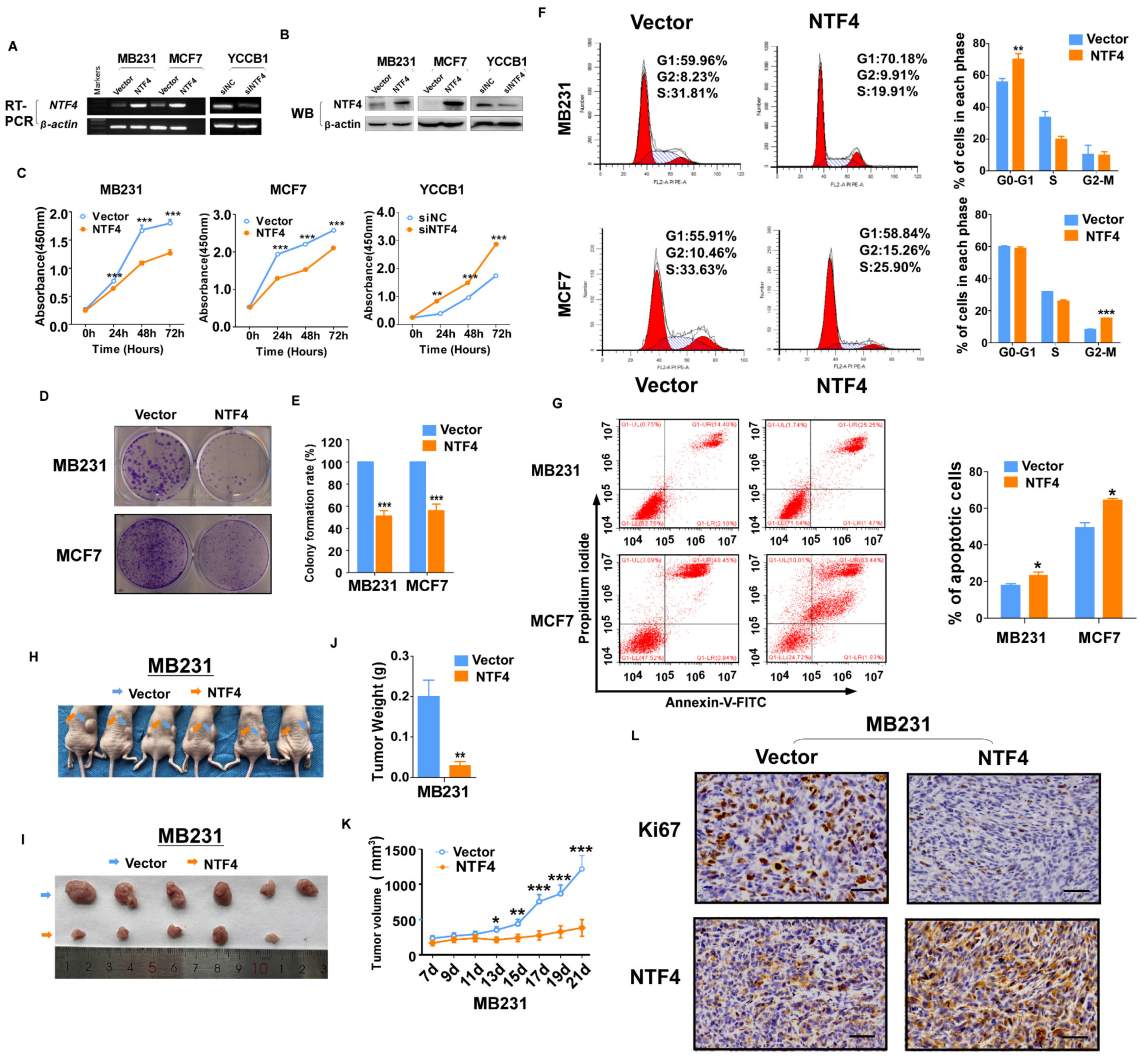
** NTF4 suppresses breast cancer cell proliferation *in vitro* and tumor growth *in vivo.* (A-E)** MDA-MB-231 and MCF7 ells stably expressing pcDNA3.1 (vector) and Flag-NTF4 (A-B) were subjected to cell proliferation assays using CCK-8 (C) and colony growth assays (D-E). **(A-C)** YCCB1 cells transfected transiently with siNC and siNTF4 (A-B) were subjected to cell proliferation assays using CCK-8 (C). **(F)** Cell cycle distribution measured in vector and Flag-NTF4 stably expressing MDA-MB-231 and MCF7 cells by flow cytometry. Representative distribution plots and histograms of alterations are shown. **(G)** Percentages of apoptotic cells in MDA-MB-231and MCF7 cells with vector and Flag-NTF4 ectopic expression were evaluated. Histograms showed the cell apoptosis alterations (right). **(H-L)** MDA-MB-231 cells stably expressing vector and Flag-NTF4 were subcutaneously injected into BALB/c nude mice (n=6). After 21 days of injected, xenograft tumors were harvested. Photographs of harvested tumors (H-I), tumor weight (J), tumor growth curves (K) and immunohistochemistry staining of NTF4 and Ki67 (L) are shown. Data are presented as the mean±SD. *p<0.05, **p<0.01, ***p<0.001.

To investigate the impact of NTF4 on the malignant phenotype of breast cancer cells, MDA-MB231 and MCF7 cell lines were constructed that stably over-expressed NTF4 (**Fig. 2A and 2B**). CCK-8 cell proliferation assays demonstrated over-expression of NTF4 to inhibit breast cancer cell proliferation (**Fig. 2C**). Colony growth assays demonstrated expression of NTF4 to decreased colony formation of breast cancer cells (**Fig. 2D and 2E**). In contrast, siNTF4 knockdown of NTF4 in the YCCB1 cell line (**Fig. 2A and 2B**) increased cell viability (**Fig. 2C**).

To investigate the mechanistic basis for growth inhibition by NTF4, cell cycle distribution assays were performed. Using the flow cytometry method, NTF4 expression blocked MDA-MB231 in G0/G1 and MCF7 in G2/M (**Fig. 2F**). Flow cytometry analysis also showed that NTF4 significantly increased the number of apoptotic breast cancer cells (**Fig. 2G**).

To investigate the effect of NTF4 on the tumorigenic capacity of breast cancer cells *in vivo*, MDA-MB-231 cells stably expressing vector or NTF4 were subcutaneously injected into BALB/c nude mice. Consistent with *in vitro* results, xenograft tumors expressing NTF4 grew slower than those expressing the empty vector (**Fig. 2H-K**). IHC was used to analyze NTF4 expression and levels of Ki-67 staining. Tumor xenografts of cells expressing NTF4 exhibited frequent cytoplasmic fragmentation as well as decreased Ki-67 staining (**Fig. 2L**). Taken together, these results suggest that NTF4 suppresses breast cancer cell proliferation *in vitro* and tumor growth *in vivo*.

### NTF4 promotes breast cancer cell migration, invasion and metastasis *in vitro* and *in vivo*

**Figure 3:**
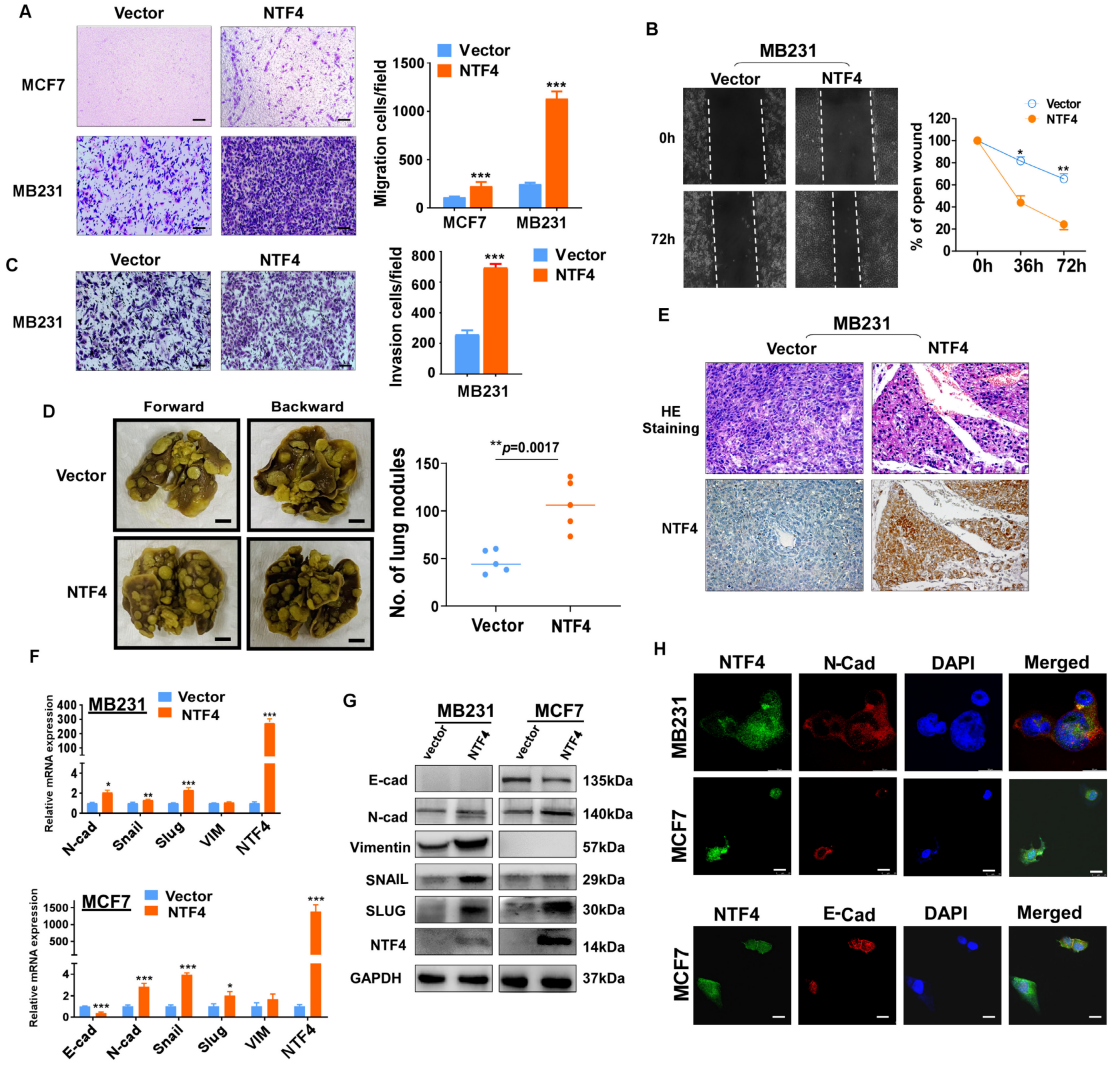
** NTF4 promotes breast cancer cell migration, invasion, EMT and metastasis. (A-C)** MDA-MB-231 and MCF7 cells stably expressing vector and Flag-NTF4 were subjected to migration assays (A), matrigel-coated invasion assays (C) and wound-healing assays (B). **(D-E)** MDA-MB-231 cells stably expressing vector and Flag-NTF4 were injected into BALB/c nude mice (n=5) through the tail vein. Representative images of lung metastasis (D, left), quantitative results of lung nodules (D, right), representative images of H.E.-stained sections of lung tissues and immunohistochemistry staining of NTF4 (E) are shown. **(F)** qRT-PCR analysis of EMT markers in MDA-MB-231 and MCF7 cells stably expressing vector and Flag-NTF4. **(G)** Immunoblotting analysis of EMT markers in MDA-MB-231 and MCF7 cells stably expressing vector and Flag-NTF4. **(H)** Immunofluorescence staining of E-cadherin and N-cadherin in MDA-MB-231 and MCF7 cells stably expressing vector and Flag-NTF4. Data are presented as the mean±SD. *p<0.05, **p<0.01, ***p<0.001.

An important hallmark of breast cancer cells is their ability to invade surrounding tissues and metastasize to distant organs 3. We next examined whether NTF4 affects migratory and invasive properties of breast cancer cells *in vitro*. Transwell^®^ assays showed MDA-MB-231 and MCF7 cells stably expressing NTF4 to have significantly increased Transwell^®^ invasion and migration with or without Matrigel (**Fig. 3A and 3C**). The results were confirmed by wound-healing assay, which showed that NTF4-expressing MDA-MB-231 had an increased wound closure rate compared to control cells (**Fig. 3B**).

To address whether NTF4 affects breast cancer metastasis *in vivo*, MDA-MB-231 cells stably expressing vector or NTF4 were injected into nude mice through the tail vein. MDA-MB-231 cells expressing NTF4 had an increased number of metastatic tumors within the lungs of nude mice (**Fig. 3D**), which was confirmed by hematoxylin and eosin (HE) staining of lung sections. The HE staining results of lung metastatic tumors showed that compared with vector-group, the lung metastatic tumors in NTF4-group were smaller in size and more in number (**Fig. 3E and Fig. S4**). Moreover, we performed IHC staining on paraffin sections of lung-metastatic tumors. The results show that, compared with vector-group, the expression of NTF4 in NTF4-group was significantly increased (**Fig. 3E**). Taken together, these results demonstrate NTF4 to promote breast cancer cell migratory and invasive behaviors *in vitro* and lung metastatic potential *in vivo*.

In addition, we have successfully constructed an MDA-MB-231-Luc metastatic cell lines that targets brain and bone, identified as MDA-MB-231-Brain-M and MDA-MB-231-Bone- M, respectively. NTF4 was assessed by qPCR and western blot for MDA-MB-231-Luc, MDA-MB-231-Brain-M, and MDA-MB-231-Bone-M (**Fig. S5A-B**). The expression of NTF4 was significantly higher in metastatic breast cancer cells, suggesting that NTF4 promotes breast cancer cell metastasis *in vivo*.

### NTF4 promotes EMT by targeting PRKDC and ANXA1

EMT plays a critical role in breast cancer invasion and metastasis 25. To investigate whether NTF4 regulates EMT, we stably expressed NTF4 in MDA-MB-231 and MCF7 cell lines. Western blot, qPCR and immunofluorescence (IF) demonstrated a downregulation of the epithelial marker (E-cadherin) and a concomitant upregulation of the mesenchymal marker (N-cadherin) in NTF4 expressing cells (**Fig. 3F-H**). But we failed to detect the expression of E-cadherin in MDA-MB-231 cells. MDA-MB-231 was reported as an E-cadherin deficient cell line 26 and the expression of E-cadherin in MDA-MB-231 was always barely to be detected 27-29. Maybe it's why we didn't detect E-cadherin in MDA-MB-231 cells.

**Figure 4:**
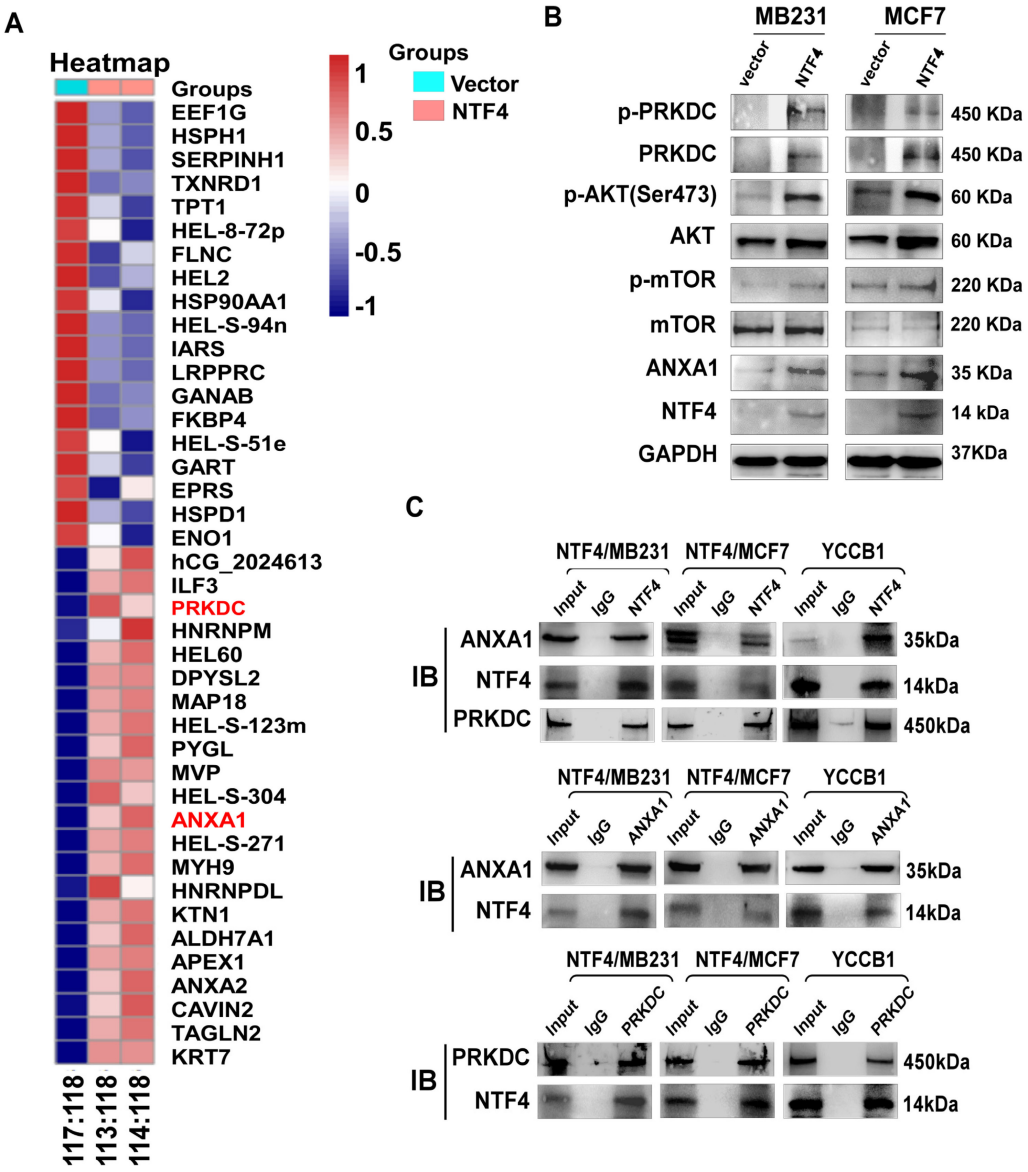
** NTF4 interacts with PRKDC and ANXA1 and activates AKT signaling. (A)** iTRAQ sequencing results in MDA-MB-231 cells stably expressing vector and Flag-NTF4. **(B)** Immunoblotting analysis of PRKDC, ANXA1 and AKT pathway related proteins in MDA-MB-231 and MCF7 cells stably expressing vector and Flag-NTF4. **(C)** Co-IP assay demonstrated NTF4 directly interact with PRKDC and ANXA1.

To assess the mechanistic basis by which NTF4 suppresses EMT, differentially expressed proteins in NTF4 stably expressing MDA-MB-231 cells were evaluated by isobaric tags for relative and absolute quantification (iTRAQ) (**Fig. 4A**). iTRAQ data showed NTF4 to significantly increase 22 proteins and to decrease 19 proteins. GSEA analysis revealed that a number of NTF4 target proteins are enriched in breast cancer PI3-kinase (PI3K) signaling pathways (**Fig. S6**). Previous studies have shown DNA-dependent protein kinase catalytic subunit (PRKDC), a member of the PI3K-like family of kinases (PIKKs), participates in the metastasis of several malignant tumors 30, 31. Moreover, studies have reported that Annexin A1 (ANXA1) enhances metastatic activity of breast cancer cells by promoting EMT 32-34. Thus, we focused on PRKDC and ANXA1 and found both proteins to be significantly increased in NTF4 stably expressing breast cancer cells, as well as constituents of an activated AKT signaling pathway (**Fig. 4B**). Moreover, co-immunoprecipitation (Co-IP) assays demonstrated NTF4 to directly interact with PRKDC and ANXA1 (**Fig. 4C**). These results demonstrate NTF4 to promote breast cancer cell EMT by binding and upregulating PRKDC and ANXA1.

### NTF4 promotes GSK-3β phosphorylation-dependent SNAIL stability through activation of the PRKDC/AKT pathway

**Figure 5:**
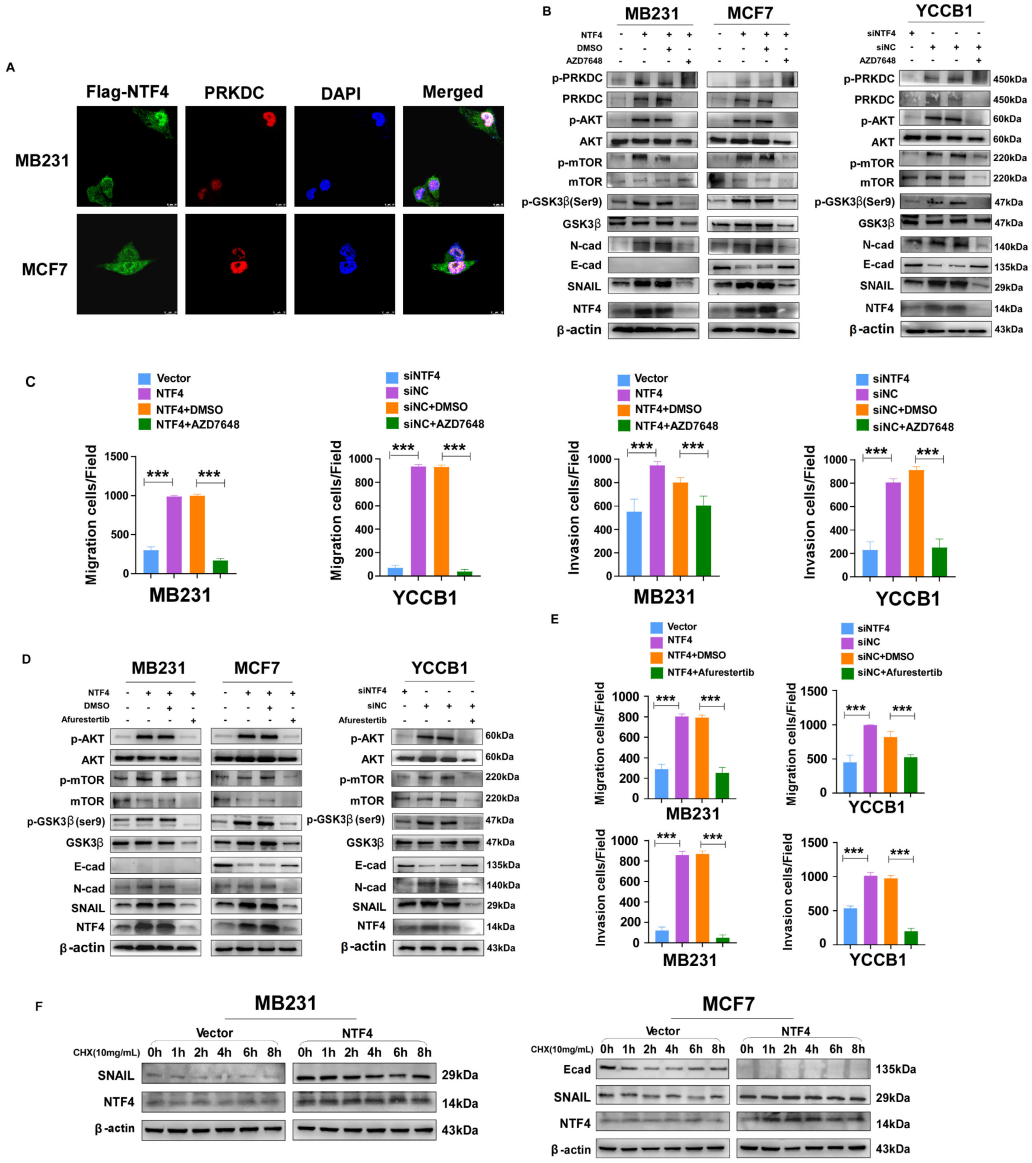
** NTF4 activates PRKDC/AKT pathway and promote GSK-3β- dependent SNAIL stability. (A)** Imunofluorescence staining of Flag and PRKDC in MDA-MB-231 and MCF7 cells stably expressing vector and Flag-NTF4.** (B-C)** MDA-MB-231 cells stably expressing vector and Flag-NTF4 or YCCB1 cells transfected transiently with siNC and siNTF4 were treated with or without 10μM PRKDC inhibitor AZD7648 for 24h and analyzed by immunoblotting (B), migration assays and matrigel-coated invasion assays (C). **(D-E)** MDA-MB-231 cells stably expressing vector and Flag-NTF4 or YCCB1 cells transfected transiently with siNC and siNTF4 were treated with or without 30μM AKT inhibitor Afurestertib for 24h and analyzed by immunoblotting (D), migration assays and matrigel-coated invasion assays (E). **(F)** MDA-MB-231 and MCF7 cells stably expressing vector and Flag-NTF4 were treated with 100μg/mL of CHX for the indicated times and then analyzed by immunoblotting.

Several studies have reported that PRKDC mediated phosphorylation of AKT Ser473 results in a 10-fold enhancement of AKT kinase activity 35, 36. Activated AKT phosphorylates GSK-3β (Ser9) promoting its ubiquitination and degradation, which increases stability of the negative transcription factor SNAIL, thereby decreasing transcription of E-cadherin 8. By using IF assays, we found that NTF4 upregulated the expression of PRKDC, and that NTF4 and PRKDC co-localized in breast cancer cells (**Fig. 5A**). By western blot, NTF4 was shown to increase expression of PRKDC and p-AKT (Ser473), which activated the AKT signaling pathway. Phosphorylation of GSK-3β (Ser9) was promoted by activated PRKDC/AKT signaling, while the expression of SNAIL was increased and E-cadherin was decreased in NTF4 over-expressing breast cancer cells (**Fig. 5B and 5D**). Rescue experiments were performed with AZD7648 (a PRKDC inhibitor) and Afurestertub (an AKT signaling inhibitor) to determine whether NTF4 activates AKT signaling through upregulation of PRKDC (**Fig. 5B and 5D**). Such upregulation would increase GSK-3β (Ser9) phosphorylation, SNAIL stability, and E-cadherin degradation. AZD7648 and Afurestertub inhibited expression of PRKDC, AKT signaling, and the mesenchymal markers (N-cadherin and SNAIL), as well as breast cancer cell migration and invasion (**Fig. 5B-E, Fig. S7 and S8**). By cycloheximide (CHX) assay, we found NTF4 to increase the stability of the negative transcription factor SNAIL (**Fig. 5F**). These results suggest that NTF4 activates the PRKDC/AKT pathway to promote phosphorylation of GSK-3β (Ser9), increasing SNAIL stability and decreasing transcription of E-cadherin.

### NTF4 promotes SNAIL stability by activation of the ANXA1/NF-κB pathway, which disrupts the interaction of GSK-3β with SNAIL

**Figure 6:**
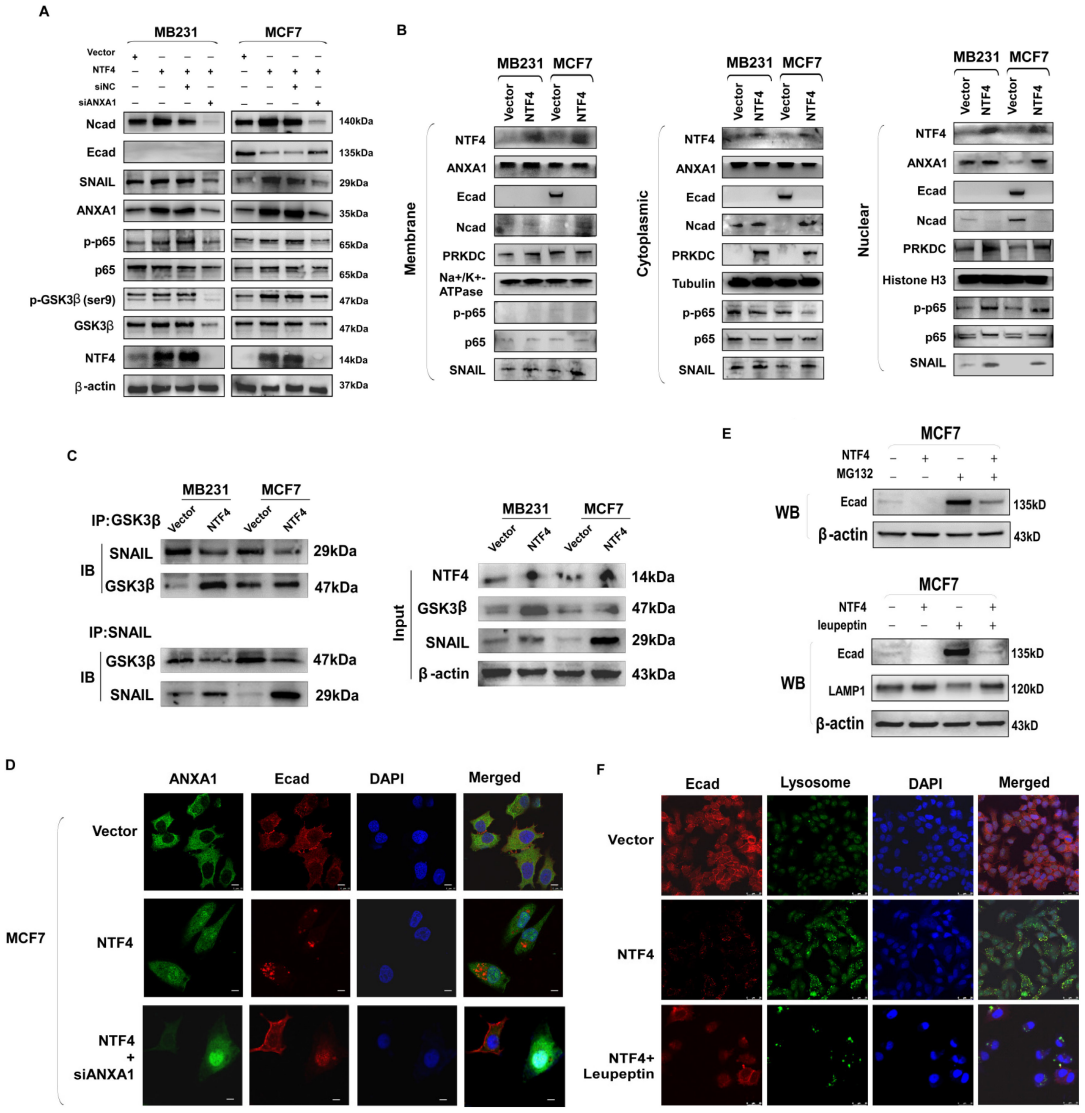
** NTF4 disrupts GSK3β-SNAIL interaction through activating ANXA1/NF-κB pathway which promotes SNAIL stability, and NTF4 promotes E-cadherin into lysosomes to degradation. (A)** MDA-MB-231 cells stably expressing vector and Flag-NTF4 were transfected transiently with siNC or siANXA1 and subjected to immunoblotting analysis. **(B)** Cytoplasmic, nuclear and membrane proteins were extracted from MDA-MB-231 and MCF7 cells stably expressing vector and Flag-NTF4 and analyzed by immunoblotting. **(C)** Lysates from MDA-MB-231 and MCF7 cells stably expressing vector and Flag-NTF4 were immunoprecipitated with an anti-GSK3β antibody or anti-SNAIL antibody, followed by immunoblotting analysis. **(D)** Imunofluorescence staining of ANXA1 and E-cadherin in MCF7 cells stably expressing vector and Flag-NTF4, and MCF7-NTF4 cells with siANXA1. **(E)** MDA-MB-231 and MCF7 cells stably expressing vector and Flag-NTF4 were treated with DMSO or 10μM MG-132 for 6h and analyzed by immunoblotting. **(F)** Imunofluorescence staining of lysosome and E-cadherin in MCF7 cells stably expressing vector, Flag-NTF4, and Flag-NTF4 with 20μM lysosome inhibitor Leupeptin for 48h.

ANXA1 directly activates NF-κB, which promotes phosphorylation of NF-κB and nuclear translocation 37. NF-κB signaling disrupts the interaction of GSK-3β with SNAIL 38, increasing the stability of SNAIL and decreasing transcription of E-cadherin 8, 39. By western blot analysis, NTF4 over-expression in MCF7 cells increased levels of ANXA1, SNAIL, and N-cadherin, as well as decreased E-cadherin (**Fig. 6A**). Moreover, western blot results showed that NTF4 increased the expression of ANXA1 and the phosphorylation of NF-κB (p-p65) (**Fig. 6A**). Further, NTF4 was shown to induce ANXA1, and p-p65 nuclear localization (**Fig. 6B**). Similarly, IF results demonstrated ANXA1 upregulation and translocation to the nucleus in NTF4 overexpressing MCF7 cells. E-cadherin underwent endocytosis and degradation (**Fig. 6D**).

Knockdown of ANXA1 was used to confirm the above. Western blots demonstrated knock-down of ANXA1 to inhibit the NF-κB pathway and the expression of mesenchymal markers (N-cadherin and SNAIL) as well as breast cancer cell migration and invasion (**Fig. 6A, Fig. S9A-B**). By western blotting and IF, knockdown of ANXA1 restored the expression of E-cadherin (**Fig. 6A and 6D**). Furthermore, by Co-IP assay, we found NTF4 to disrupt the interaction of GSK-3β with SNAIL (**Fig. 6C**). After CHX treatment of breast cancer cells, NTF4 was found to increase the stability of SNAIL (**Fig. 5F**). Treatment of breast cancer cells with MG132 demonstrated that NTF4 reduced the generation of E-cadherin, which is consistent with qPCR results (**Fig. 6E and 3F**). Taken together, these results suggest that NTF4 activates the ANXA1/NF-κB pathway to disrupt interaction of GSK-3β with SNAIL, which increases SNAIL stability and decreases E-cadherin transcription.

### NTF4 promotes lysosomal degradation of E-cadherin

Protein down-regulation may be due to decreased production and/or increased degradation. To determine whether NTF4 increases the degradation of E-cadherin, cells were treated with CHX. In those cells, NTF4 was found to promote degradation of E-cadherin (**Fig. 5F**). There are three main protein degradation pathways: the ubiquitin proteolytic pathway, the lysosomal pathway, and the caspase pathway. *In vitro* ubiquitination assays showed that NTF4 did not increase the ubiquitination level of E-cadherin (data not show). IF and western blot were used to assess E-cadherin degradation. NTF4 was found to increase the number of lysosomes and the degradation of E-cadherin within lysosomes. E-cadherin levels were partially restored after leupeptin (lysosomal inhibitor) treatment (**Fig. 6E and 6F**). Likewise, western blot results showed NTF4 to upregulate the expression of the lysosomal marker, LAMP1, and to downregulate the expression of E-cadherin. Leupeptin partially restored E-cadherin expression (**Fig. 6E**). These results suggest that NTF4 promotes the degradation of E-cadherin through the lysosomal pathway.

### NTF4 promotes cellular apoptosis by promotion of ANXA1 nuclear translocation

**Figure 7:**
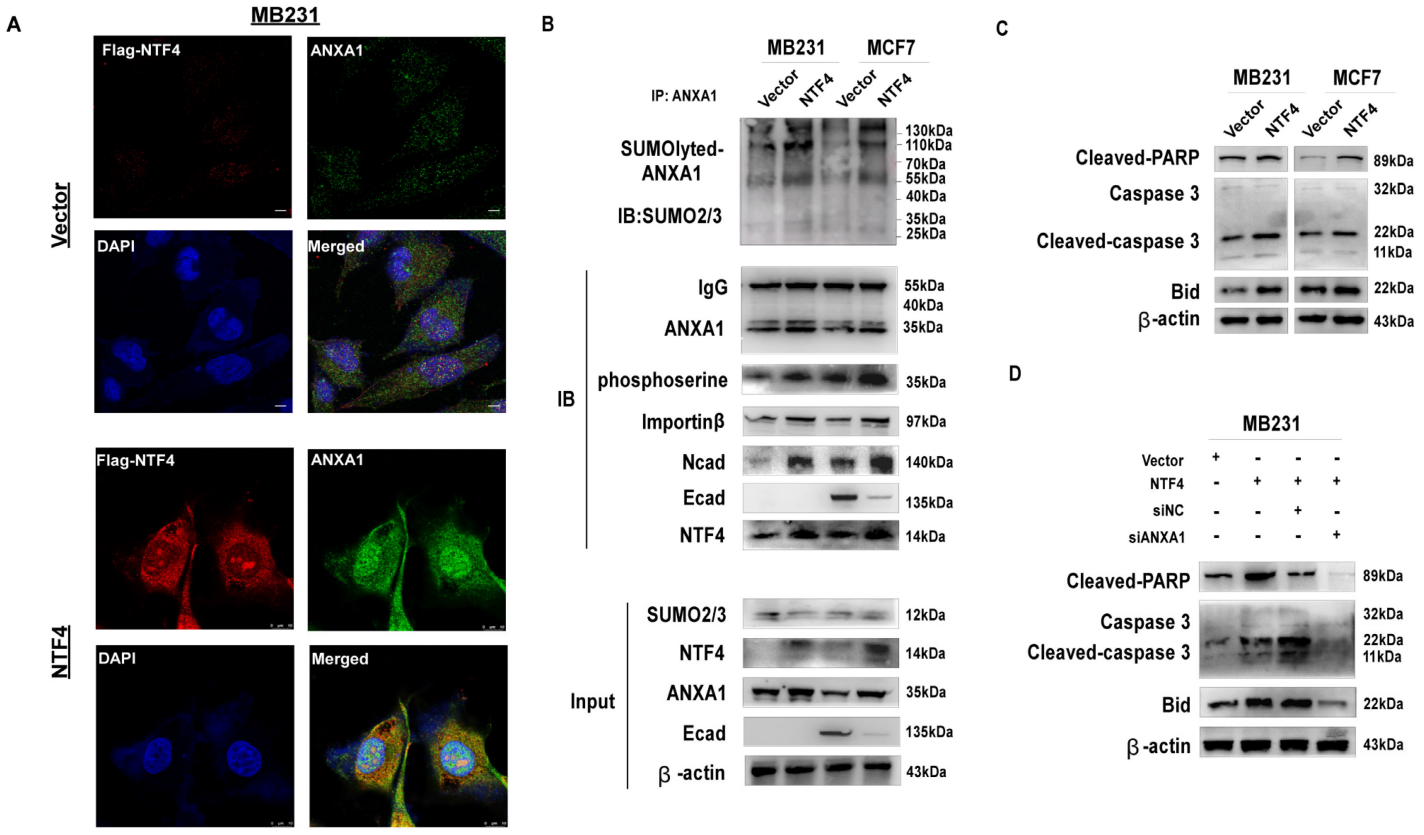
** NTF4 promotes cell apoptosis through mediating ANXA1 nuclear translocation. (A)** Imunofluorescence staining of Flag and ANXA1 in MDA-MB-231 cells stably expressing vector and Flag-NTF4. **(B)** Lysates from MDA-MB-231 and MCF7 cells stably expressing vector and Flag-NTF4 were immunoprecipitated with an anti-ANXA1 antibody, followed by immunoblotting analysis. **(C)** Immunoblotting analysis of Caspase-3 apoptosis cascade protein in MDA-MB-231 and MCF7 cells stably expressing vector and Flag-NTF4. **(D)** Immunoblotting analysis of Caspase-3 apoptosis cascade protein in MDA-MB-231 cells stably expressing vector and Flag-NTF4, and MDA-MB-231- NTF4 cells with siNC or siANXA1.

By the IF assay, NTF4 was shown to promote nuclear translocation of ANXA1 **(Fig. 6D and 7A)**. Western blots demonstrated NTF4 to promote nuclear translocation of ANXA1 by increasing sumoylation and serine phosphorylation of ANXA1. Further, NTF4 enhanced the interaction between ANXA1 and nuclear transfer factor importin β **(Fig. 7B)**. Moreover, importin β-dependent nuclear transcription of ANXA1 promoted the expression of pro-apoptotic factor, Bid, and activated the caspase3 signaling pathway, which resulted in breast cancer cellular apoptosis** (Fig. 7C)**. Western blot results demonstrated knockdown of ANXA1 to inhibit Bid and caspase3 signaling **(Fig. 7D)**. These results suggest that NTF4 promotes cellular apoptosis through promotion of ANXA1 nuclear translocation.

## Discussion

In this study, we present several unexpected findings that demonstrate important but complex roles for NTF4 in malignant progression and metastasis of breast cancer.

Firstly, NTF4 plays both anti-tumorigenic and pro-metastatic roles in breast cancer progression. Neurotrophins and their receptors have been previously demonstrated to participate in the tumorigenesis of multiple carcinomas 9-15 including breast cancer 16-19. However, the precise role of neurotrophins in cancer is essentially unknown. Herein, we report that NTF4 plays a dual role in the malignant progression of breast cancer. In support of our results, a few cancer-relevant signaling molecules have been shown to exert dual roles in cancer development and progression. For example, the Ski-related novel protein N (SnoN), a negative regulator of transforming growth factor β (TGFβ) signaling, has been shown to promote mammalian tumorigenesis but also to inhibit EMT and tumor metastasis 40. Another example is F-box only protein 22 (FBXO22), which promotes breast cancer cell proliferation, but suppresses EMT, breast cancer invasion, and metastasis through GSK3β phosphorylation-dependent degradation of SNAIL 2.

In this study, we found NTF4 inhibited cell proliferation as well as promoted cellular apoptosis *in vitro* and inhibited xenograft tumorigenicity *in vivo*. Surprisingly, NTF4 promoted EMT, cell motility and invasiveness *in vitro* and metastatic lung colonization *in vivo*. Based on those investigations, we hypothesized a role for NTF4 in the inhibition of primary tumor colonies by decreasing tumor cell viability and growth at an early stage of tumorigenesis, which may lead to the smaller tumor volume observed in clinic, presenting a “false impression of early tumor”.

Most researches have assumed that the spread, or metastasis, of tumors typically occurs later in the disease process. But many cancers are likely to have spread from the site where they first formed to other parts of the body long before the original tumor can be detected by current screening tests, the new study finds was originally published by the National Cancer Institute. Metastasis begins very early during tumor development—when the primary tumor is smaller than the tip of a sharpened pencil, in other word, some tumors may be “born to be bad”.

Based on differential NTF4 expression in distinct breast tissues, our findings demonstrate NTF4 was downregulated in primary breast tumors, but upregulated in normal breast tissues and in metastatic tumors, which suggested that NTF4 may play a multifaceted role in breast cancer progression, and metastases. With primary tumor progress to invasion and metastasis, NTF4 expression increases through an unknown mechanism that allows tumor cells to undergo EMT, invasion and metastasis. Interestingly, HE staining results of lung metastatic tissues* in vivo* showed that compared with vector-group, the lung metastatic tumors in NTF4-group were smaller in size and more in number. As well as, clinical data showed high levels of NTF4, especially in the early stage, to be associated with poor clinical outcomes, supporting the notion that metastasis, rather than primary cancer, was the major determinant of breast cancer mortality for patients. What's more, clinical data revealed NTF4 may be a new potential biomarker for predictive early breast cancer risk for metastasis.

Secondly, NTF4 is a new regulator of breast cancer EMT and cancer progression, which targets PRKDC (DNA-PKcs) and ANXA1 and activate AKT and NF-κB pathways. PRKDC belongs to the PI3K-related kinase family which is involved in the cellular DNA damage and repair response, mediating DNA non-homologous end joining. The enzyme has two components, one of which is PRKDC encoded by the DNA-PK gene. As an important component of DNA-PK, PRKDC is not only involved in the DNA damage response, it also promotes the migration and invasion of tumor cells and the expression and secretion of EMT-related proteins 41, 42. Phosphorylation at Ser473 is required for full activation of AKT, with Ser473 phosphorylation mediated by PRKDC 35, 36. ANXA1 is located in membranes and has the ability to bind phospholipids, regulating cell adhesion. One study has reported ANXA1 to directly combine with E-cadherin, enhancing the metastatic activity of breast cancer cells by promoting EMT 43. ANXA1 directly activates NF-κB by interacting with and stabilizing the NEMO-RIP1 complex, which is important for NF-κB phosphorylation and nuclear translocation 37. Interestingly, ANXA1 was downregulated in primary breast tumors, but upregulated in normal breast tissues and in metastatic tumors, and breast cancer patients with high levels of ANXA1 have a poorer clinical prognosis 44-47, which is consistent with the expression changes of NTF4 in distinct breast tissues. We hypothesized that NTF4 combined with ANXA1 may be combined predictive and therapeutic targets for breast cancer.

Activated AKT phosphorylates GSK-3β, which promotes its ubiquitination and degradation, increasing the stability of the negative transcription factor SNAIL. SNAIL decreases transcription of the transmembrane protein E-cadherin, which forms adhesions between adjacent cells, permitting their detachment 8. NF-κB signaling can activate SNAIL directly 38. The AKT pathway inhibits SNAIL phosphorylation by GSK-3β 48, with NF-κB signaling disrupting GSK-3β-SNAIL 49. Both these mechanisms increase SNAIL stability. SNAIL represses transcription of E-cadherin and decreases E-cadherin in approximately half of all tumors 8, 39.

In this study, we found NTF4 to promote breast cancer EMT, invasion, and migration. Further, NTF4 was found to promote EMT through activation of PRKDC/AKT and ANXA1/NF-κB signaling. Consistent with previous reports, activated AKT phosphorylates GSK-3β (Ser9), and although not involved in proteolytic removal, stabilization of SNAIL enhances the repression of E-cadherin expression. Moreover, activated NF-κB increases SNAIL stability by upregulating the expression of SNAIL and the degradation of E-cadherin. Moreover, inhibition of PRKDC/AKT signaling and ANXA1 effectively rescues NTF4-mediated breast cancer metastasis and invasion. These results suggest that NTF4 exerts its pro-metastasis function through activation of PRKDC- and ANXA1-mediated EMT. Interestingly, we observed that NTF4 expression was downregulated when using the inhibitors AZD7648 and Afurestertib, and speculated whether NTF4/PRKDC/AKT directly formed a positive feedback loop regulation. That is, NTF4 activates the PRKDC/AKT signalling, and the activated PRKDC/AKT signalling then promotes NTF4 expression. Therefore, when the inhibitors are used to inhibit the expression of PRKDC or the activity of AKT pathway, NTF4 is down regulated. Similarly, such a positive feedback loop is also formed between NTF4 and ANXA1.

Thirdly, NTF4 exert its anti-proliferative effect by increasing the phosphoserine and sumoylation levels of ANXA1 and the expression of nuclear transfer factor importin β, important to ANXA1 import into and retention within the nucleus. Moreover, ANXA1 nuclear translocation promotes expression of the pro-apoptotic Bid gene, which activates the caspase-3 apoptosis cascade, ultimately resulting in breast cancer cell apoptosis. ANXA1 subcellular localization ultimately determines cellular fate. In inflammatory diseases and the immune response system, treatment with exogenous ANXA1 mimetic peptides decreases the expression of pro-inflammatory mediators, which attenuate the infiltration of inflammatory cells 42. Extracellular ANXA1 promotes stem cell differentiation 50 and reduces cancer cell growth and aggressiveness 51. ANXA1 nuclear translocation also induces cellular apoptosis by interaction with p53 and p65 52. In this study, we found NTF4 to promote ANXA1 nuclear translocation. Thus, we speculated that overexpression of NTF4 would induce breast cancer cell apoptosis by ANXA1 nuclear translocation. Further, ANXA1 has been shown to directly interact with E-cadherin 43. Combined with the above, we speculated that breast cancer cells over-expressing NTF4 would increase ANXA1 nuclear translocation, which would promote E-cadherin endocytosis and facilitation of E-cadherin entry into lysosomes for degradation. In the future, we will continue to carry out experiments to verify this speculation.

Because sumoylation regulates the translocation of many proteins between the nucleus and cytoplasm 53-55, we questioned whether NTF4 mediated ANXA1 sumoylation regulates its subcellular localization. In the study, we found ANXA1 to interact with SUMO2/3 and that sumoylation of ANXA1 was greatly increased after NTF4 over-expression. Furthermore, nuclear translocation of ANXA1 is associated with a phosphorylation process (tyrosine and possibly threonine and serine) 53, with our results demonstrating NTF4 to increase serine phosphorylation of ANXA1.

Importin α and -β are considered classic nuclear envelope transporters. In this study, we found that NTF4 over-expression increases ANXA1 binding by importin β, which promotes ANXA1 nuclear localization. A previous study reported that importin β-dependent nuclear ANXA1 translocation is involved in oxygen-glucose deprivation and reperfusion (OGD/R)-induced neuronal apoptosis. As a cofactor, ANXA1 binds p53 in the nucleus and upregulates p53 transcriptional activity, thereby promoting pro-apoptotic Bid expression and caspase-3 apoptosis pathway activation, which result in neuronal apoptosis after ischemic stroke 56. Another study reported ANXA1 to interact with Bid, whereby it serves as an apoptosis “regulator” within the nucleus 57. In this study, we found NTF4 to promote nuclear translocation of ANXA1 by increasing the interaction between ANXA1 and importin β, which upregulates the expression of the pro-apoptotic protein, Bid, and activates the caspase-3 apoptosis pathway, resulting in cellular apoptosis. Furthermore, after nuclear translocation of ANXA1, the complex formed with E-cadherin dissociates and E-cadherin enters lysosomes for degradation.

In summary, NTF4 was shown to play both pro-metastatic and anti-tumorigenic roles in breast cancer progression. NTF4 promotes the EMT process by activation of PRKDC/AKT and ANXA1/NF-κB signaling, increasing SNAIL stability. SNAIL decreases transcription of E-cadherin and promotes the degradation of E-cadherin through lysosomal degradation. As well, NTF4 suppresses the proliferation of breast cancer cells by increasing the phosphoserine and sumoylation level of ANXA1 and the interaction of ANXA1 with importin β. In this manner, ANXA1 is imported and retained in the nucleus, with subsequent upregulation of Bid and activation of the caspase-3 apoptosis cascade, resulting in cell apoptosis. In other words, NTF4 can induce apoptosis of precancerous cells and inhibit the proliferation of cancer cells. However, when tumors progress, NTF4 promotes EMT and invasion and metastasis of tumor cells. Moreover, the expression changes of NTF4 and ANXA1 in different breast tissues are consistent. Clinical data showed high levels of NTF4, especially in the early stage, to be associated with poor clinical outcomes. Hence, NTF4 or NTF4 combined with ANXA1 may be biomarkers for breast cancer prognosis, especially for early stage breast cancer patients at risk for metastasis. These new findings provide mechanistic insight into the functional role of NTF4 in the regulation of breast cancer development and progression and may have specific clinical relevance.

## Materials and methods

### Cell lines and reagents

Breast cancer cell lines (MDA-MB231, MCF7, YCCB1, etc.), and immortalized human mammary epithelial cell lines (MCF-10A, HMEC) were obtained from the American Type Culture Collection (ATCC, Manassas, VA, USA) or collaborators. These cell lines were cultured in RPMI 1640 medium (Gibco-BRL, Karlsruhe, Germany) or DEME medium supplemented with 10% fetal bovine serum (Gibco-BRL) and 1% penicillin-streptomycin (Gibco-BRL) according to standard protocols. siRNAs used for knocking down NTF4 and ANXA1 were purchased from Origene according to its Application Guide. The PRKDC inhibotor AZD7648 (10μM) and AKT inhibitor Afurestertib (30μM) were performed according to the manufacturers' recommendations (MedChemExpess, New Jersey, USA).

### Tissue specimens

The breast cancer tissues, paired adjacent non-tumor tissues, normal breast tissues and metastatic tissues were all obtained from the First Affiliated Hospital of Chongqing Medical University (CQMU 2016-75) and RNA was extracted for RNA-seq screening. All samples were reviewed and subjected to histological as reported previously 58.

### DNA and RNA extraction

Genomic DNA was extracted from cell lines and tissues samples with a QIAamp DNA mini kit according to the manufacturer's instructions (Qiagen, Hilden, Germany). Total RNA was extracted from cell lines and tissue samples with TRIzol reagent (Invitrogen, Carlsbad, CA, USA) 59. The concentrations of DNA and RNA samples were measured through NanoDrop^®^ 2000 spectrophotometry (Thermo Fisher Scientific, Waltham, MA). The sample quality was determined by gel electrophoresis, and samples were stored at -80°C.

### Reverse transcription (RT)-PCR and real-time PCR (qRT-PCR)

Genomic RNA and total DNA were isolated from cell lines and tissues using TRI Reagent® (Molecular Research Center, Cincinnati, OH, USA) and DNAzol® reagent (Invitrogen, Rockville, MD), respectively, according to the manufacturer's instructions. The concentration of the samples was determined by spectrophotometry using a NanoDropTM 2000 (Thermo scientific). Reverse transcription of RNA was using GoScriptTM Reverse Transcription (Promega, Madison, WI, USA) 59. Semi-quantitative RT-PCR was carried out with Go-Taq DNA polymerase (Promega, Madison, WI, 487 USA) under the conditions detailed in a previous study 59, 60. Real-time PCR (qRT-PCR) was performed with SYBR (Promega) on an HT7500 Real-Time PCR system (Applied Biosystems, Foster, CA, USA) according to the instrument manual 59. The relative expression was estimated with the 2^-△Ct^ method 59, and all assays were performed in triplicate. *GAPDH* or *Actin* was amplified as a loading control for RNA integrity. All primers and reaction systems are listed in Table 1.

### Plasmids and generation of stable cell lines

The full length NTF4 gene was inserted into a pcDNA3.1(+) framework plasmid, and the plasmid was recombined as in previous work. Lipofectamine 2000 (Invitrogen, Carlsbad, CA, USA) were used for transfection following the manufacturers' instructions. MDA-MB231 and MCF7 were transfected with NTF4 plasmids and filtrated with G418 to establish stably overexpressing-NTF4 cell lines. The pcDNA3.1-empty plasmid was transfected into generated control cell lines. RT-PCR and Western blot were performed to confirm ectopic expression of NTF4.

### Cell proliferation assay

Cell viabilities were evaluated at 0, 24, 48, and 72 h with the Cell Counting Kit-8 (Beyotime, Shanghai, China) 60. The colony formation assay was performed as previously described 60. Ectopic NTF4-expressing cells or vector-transfected cells were plated in six-well plates (800 cells/well). Surviving colonies (≥50 cells per colony) were visualized with gentian violet (Beyotime Institute of Biotechnology, Jiangsu, China) staining and counted. All experiments were repeated three times.

### Mobility assays

Cell mobility was assessed with scratch wound healing assays. MDA-MB231 and MCF7 cells with stable NTF4 expression were cultured in six-well plates until confluent, and the vector-transfected cells were used as controls. The cell layers were carefully wounded with pipette tips, and cell migration distance was measured at various times through phase contrast microscopy (Leica DMI4000B, Milton Keynes, Bucks, UK).

*In vitro* Transwell^®^ assays were carried out as described previously 60. The Transwell^®^ chambers (8-μm pore size, BD Sciences, Bedford, MA, USA) were used with or without Matrigel (BD Biosciences, San Jose, CA, USA) to measure cell migration and invasion, respectively. Cells on the lower membrane surface were counted after fixation and staining. All experiments were independently repeated three times.

### Cell cycle and apoptosis analyses

Cell cycle arrest and apoptosis were assayed by flow cytometry as described previously 59. For cell cycle and apoptosis analysis, NTF4 stably transfected cells (MDA-MB231 and MCF7) were used. Cells were harvested with trypsin, fixed with ice-cold 70% ethanol and stained with propidium iodide to assay for cell cycle distribution. Apoptosis was assayed with Annexin V-fluorescein isothiocyanate and propidium iodide staining according to the kit manufacturer's protocol. Data were analyzed with a CELL Quest kit (BD Biosciences). All experiments were independently repeated in triplicate.

### Immunofluorescence (IF)

MDA-MB231 and MCF7 cells were seeded in 24-well plates on coverslips and allowed to grow overnight; the cultures were then infected transiently with NTF4. Coverslips were stained through indirect immunofluorescence double staining, as described previously 60. The staining of lysosome was performed according to the manufacturers' recommendations (Beyotime Institute of Biotechnology). For assessing the degradation pathway of proteins, cells were treated with 20μM leupeptin (lysosome inhibitor) for 48h and then were fixed and stained. Briefly, cells were incubated with primary antibodies against NTF4 (#sc-365444, Santa Cruz Biotechnology, Santa Cruz, CA, USA) , Flag (#14793, Cell Signaling Technology), E-cadherin (#14472, Cell Signaling Technology), N-cadherin (#13116, Cell Signaling Technology) and PRKDC (#12311, Cell Signaling Technology; sc-5282, Santa Cruz), ANXA1 (sc-12740, Santa Cruz), and then incubated with Alexa Fluor® 594 and 488 (Invitrogen Life Sciences, Carlsbad, CA, USA) secondary antibodies against mouse or rabbit IgG. Cells were then counterstained with 4′, 6-diamidino-2-phenylindole and imaged with a confocal laser scanning microscope (Leica Microsystems CMS GmbH Am Friedensplatz 3, 68165 Mannheim Germany). All experiments were independently repeated three times.

### Immunohistochemistry (IHC)

Samples from both patients and animals were studied following a previously published protocol 60. Anti-NTF4 was obtained from Santa Cruz (sc-365444, Santa Cruz). Sections were incubated with primary antibody (1:100 dilution) overnight at 4°C and then with secondary antibody (1:2000 dilution) at 37°C for 30 min. Then, signals were visualized with diaminobenzidine and the slides were counterstained with hematoxylin. After the final staining, the samples were scanned using an IHC scanner (PANNNORAMIC MIDI, Budapest, Hungary) and processed by CaseViewer (v2.2.0.85100, Budapest, Hungary). IHC scores were determined according to the staining intensity (0: < 5%; 1: 5%-25%; 2: 26%-50%; 3: 51%-75%; 4: >75%). An overall score was derived by multiplying the intensity and percentage scores.

### *In vivo* experiments

BALB/c nude mice (4-6 weeks old, female, 15-19g) were Beijing Vital River Experimental Animal Technology Co. Ltd. and housed in the Experimental Animal Center of Chongqing Medical University, China. The study was approved by Medical Laboratory Animal Welfare and Ethics Committee of the First Affiliated Hospital of Chongqing Medical University (Approval notice: #2016-75) and the methods were carried out in accordance with the approved guidelines. The mice were randomly divided to groups (n = 6 for each group). MDA-MB-231 stably expressing NTF4 and vector cells (5×10^6^ cells in 0.1ml PBS per mouse) were injected subcutaneously into the lower backs of BALB/c nude mice. Tumors were examined every 3 days; the length, width, and thickness were measured with calipers, and tumor volumes were calculated using the equation (Length × Width^2^)/2. 21 days after injection, the animals were euthanized, and the tumors were excised, weighed, paraffin-embedded and subjected to staining assays.

For metastasis experiments, MDA-MB-231 stably expressing NTF4 and vector cells (2×10^6^ cells in 0.1ml PBS per mouse) were injected into the tail vein of 10 female BALB/c nude mice (4-6 weeks old, 15-19g) which were randomly divided into two groups (n=5 for each group). After 27 days of injection, the animals were euthanized (when the status of the mouse becomes worse), and the intact lung tissues were isolated from the mice. The tissue sections were stained with hematoxylin and eosin and IHC. The numbers of metastatic cancer nests were counted and photographed.

### Western blot

Western blot was performed as described previously 60. Subcellular fractionation was performed using the subcellular protein fractionation kit (Thermo Scientific Pierce) according to the manufacturer's instructions. The lysosome inhibitor Leupeptin was performed according to the manufacturers' recommendations (Beyotime Institute of Biotechnology). The primary antibodies as following: NTF4 (sc-365444, Santa Cruz), Flag (G188, Abm, Canada), PRKDC (#38168, Cell Signaling Technology; sc-5282, Santa Cruz), p-PRKDC (#68716, Cell Signaling Technology), AKT (#9272, Cell Signaling Technology), p-AKT (Ser473) (#4060, Cell Signaling Technology), mTOR (#2983, Cell Signaling Technology), p-mTOR (#5536, Cell Signaling Technology), GSK-3β (sc-377213, Santa Cruz), p-GSK-3β (Ser9) ( sc-373800, Santa Cruz), p65 (sc-8008, Santa Cruz), p-p65 (sc-136548, Santa Cruz), E-cadherin (#14472, Cell Signaling Technology), N-cadherin (#13116, Cell Signaling Technology), SNAIL (#3897, Cell Signaling Technology), ANXA1 (sc-12740, Santa Cruz), Histone H3 (#4499, Cell Signaling Technology), α-Tubulin (#3873, Cell Signaling Technology), Na^+^/K^+^-ATPase (#3010, Cell Signaling Technology), SUMO2/3 (#4917, Cell Signaling Technology), Importin β (#51186, Cell Signaling Technology), BID ( #2002, Cell Signaling Technology), Caspase3 (#14220, Cell Signaling Technology ), Cleaved-caspase3 (#9664, Cell Signaling Technology), Cleaved-PARP (#5625, Cell Signaling Technology), LAMP1 (#9091, Cell Signaling Technology), and β-actin (sc-47778, Santa Cruz, loading control). The dilution of primary and secondary antibodies was performed according to the manufacturers' recommendations. And Restore™ Western Blot Stripping Buffer (#21059, Thermo Fisher) was used to strip protein mixture on the PVDF membranes according to manufacturer's protocol. Goat Anti-Mouse IgG, Peroxidase-Conjugated (BL001A; Biosharp, Hefei, China) and Goat Anti-Rabbit IgG, Peroxidase-Conjugated (BL003A; Biosharp, Hefei, China) were used as secondary antibodies. The chemiluminescence kit (Amersham Pharmacia Biotech, Piscataway, NJ) was used to visualize protein bands in a Gel Imager System (FX5, Vilber Lourmat). All assays were independently repeated three times.

### Co-immunoprecipitation (Co-IP)

Co-IP was performed to confirm protein-protein binding. MilliporeSigma™ PureProteome™ Protein A/G Mix Magnetic Bead System (#LSKAGAG10, Fisher Scientific, USA) and detailed protocol was published on the former report 60. The primary antibodies as following: NTF4 (sc-365444, Santa Cruz), PRKDC (sc-5282, Santa Cruz), ANXA1 (sc-12740, Santa Cruz), GSK-3β (sc-377213, Santa Cruz), SNAIL (#3897, Cell Signaling Technology), and β-actin (sc-47778; Santa Cruz; loading control). The Co-IP complexes were analyzed by SDS-PAGE and immunoblot. And anti-mouse IgG (#A25012, Abbkine) was used as second antibodies for Co-IP complexes detection and the Co-IP complexes.

### CHX and MG-132 assays

For assessing the half-life of proteins, cells were treated with 100 μg/mL cycloheximide (CHX) and then harvested at indicated time points for immunblotting analysis. For analysis the degradation pathway of proteins, cells were treated with 10μM MG-132 (proteasome inhibitor) for 6h and then harvested for immunblotting.

### Statistical analysis

Statistical analyses involved Student's t-test, chi-squared test, One-way ANOVA, Fisher's exact test and Kaplan-Meier analysis with SPSS25.0 software (version 25.0, IBM, SPSS, Chicago, USA) or GraphPad Prism 8 (GraphPad Software, Inc., La Jolla, CA). Data are shown as mean ± SD from at least three independent experiments. *p*<0.05 was considered statistically significant.

## Supplementary Material

Supplementary figures.

## Figures and Tables

**Table 1:** List of PCR primers used in this study

PCR	Primer	Sequence (5'-3')	Product size (bp)	Annealing temperature (°C)
	*NTF4F*	GCAAGGCTGATAACGCTGAG	119bp	60
	*NTF4R*	CAATGCCCGCACATAGGACT		
	*GAPDHF*	CCAGCAAGAGCACAAGAGGAA	114bp	55
	*GAPDHR*	GGTCTACATGGCAACTCAAGG		
	*E-cadherinF*	TACACTGCCCAGGAGCCAGA	103bp	60
	*E-cadherinR*	TGGCACCAGTGTCCGGATTA		
	*N-cadherinF*	CGAATGGATGAAAGACCCATCC	174bp	60
	*N-cadherinR*	GGAGCCACTGCCTTCATAGTCAA		
	*SNAILF*	CGCGCTCTTTCCTCGTCAG	181bp	60
	*SNAILR*	CGCGCTCTTTCCTCGTCAG		
	*SLUGF*	CGAACTGGACACACATACAGTG	87bp	60
	*SLUGR*	CTGAGGATCTCTGGTTGTGGT		
	*VimentinF*	GACCAGCTAACCAACGACAA	150bp	60
	*VimentinR*	GTCAACATCCTGTCTGAAAGAT		

## Abbreviations

NTF4: neurotrophin 4
CCK8: cell counting kit-8
Co-IP: co-immunoprecipitation
CHX: cycloheximide
ER: estrogen receptor
PR: progesterone receptor
HER2: human epidermal growth receptor 2
TNBC: triple-negative breast cancer
EMT: epithelial-mesenchymal transition
IB: Immunoblot
IF: immunofluorescence
IHC: immunohistochemistry
qRT-PCR: real-time PCR
RNA-seq: RNA sequences
TCGA: the cancer genome atlas
