# Supplementary material for: NTF4 plays a dual role in breast cancer in mammary tumorigenesis and metastatic progression
Source: Int J Biol Sci. 2023 Jan 1;19(2):641–57. doi: 10.7150/ijbs.79435 (PMC9830504; doi:10.7150/ijbs.79435)
Supplement: Supplementary file 1 — Supplementary figures. [file ijbsv19p0641s1.pdf]

Supplementary figure

Figure S1. NTF4 expression is associated with the prognosis of patients with breast cancer

(A) RNA-seq screening of the expression of NTF4 in normal breast tissues (BN), adjacent normal tissues (BA), breast cancer tissues (BrCa), and metastatic breast tissue (BrCa-M). (B) Kaplan-Meier curves of OS and DMFS of breast cancer patients with high or low NTF4 expression. Data are presented as the mean±SD. (C) The area under the ROC curve of NTF4 in breast cancer.

Figure S2. Kaplan-Meier curves of OS and RFS of breast cancer patients with high or low NTF4 expression in different molecular subtypes. Data are presented as the mean±SD. (https://kmplot.com/analysis/)

Figure S3. Kaplan-Meier curves of OS and RFS of breast cancer patients with high or low NTF4 expression in different pathological grading. Data are presented as the mean±SD. (https://kmplot.com/analysis/)

Figure S4. Representative images of H.E.-stained sections of lung metastatic tissues are shown.

Figure S5. NTF4 promotes breast cancer cell metastasis and in vivo

(A) The animal model of metastasis of MDA-MB-231-Luc cells (Ctrl) which targeting brain metastases and bone metastases were constructed. Brain-M5 refers to the selection of the 5th passage of MDA-MB-231-Luc cells directed to the brain. Bone-M4 refers to the selection of the 4th passage of MDA-MB-231-Luc cells directed to the bone. qRT-PCR analysis of NTF4 in MDA-MB-231-Luc (Ctrl), MDA-MB-231-Luc-Brain-M5 (Brain-M5) and MDA-MB-231-Luc-Bone-M4 (Bone-M4). (B) Western blot analysis of NTF4 in MDA-MB-231-Luc (Ctrl), MDA-MB-231-Luc-Brain-M5 (Brain-M5) and MDA-MB-231-Luc-Bone-M4 (Bone-M4).

Figure S6. NTF4 promotes target genes enriching in PI3K signaling in breast cancer

GSEA analysis revealed that lots of target proteins of NTF4 are enriched in PI3K signaling pathway in breast cancer.

Figure S7. NTF4 promotes EMT by activating PRKDC/AKT pathway

MDA-MB-231 cells stably expressing vector and Flag-NTF4 were treated with or without 10μM PRKDC inhibitor AZD7648, 30μM AKT inhibitor Afurestertib for 24h and analyzed by migration assays and matrigel-coated invasion assays.

Figure S8. NTF4 promotes EMT by activating PRKDC/AKT pathway

YCCB1 cells transfected transiently with siNC and siNTF4 were treated with or without 10μM PRKDC inhibitor AZD7648, 30μM AKT inhibitor Afurestertib for 24h and analyzed by migration assays and matrigel-coated invasion assays.

Figure S9. NTF4 promotes EMT by activating ANXA1 pathway

MDA-MB-231 cells stably expressing vector and Flag-NTF4 were transfected transiently with siNC or siANXA1 and analyzed by migration assays and matrigel-coated invasion assays. (B) MDA-MB-231 cells stably expressing vector and Flag-NTF4 transfected transiently with siNC and siANXA1 and analyzed by migration assays and matrigel-coated invasion assays.

Figure S1

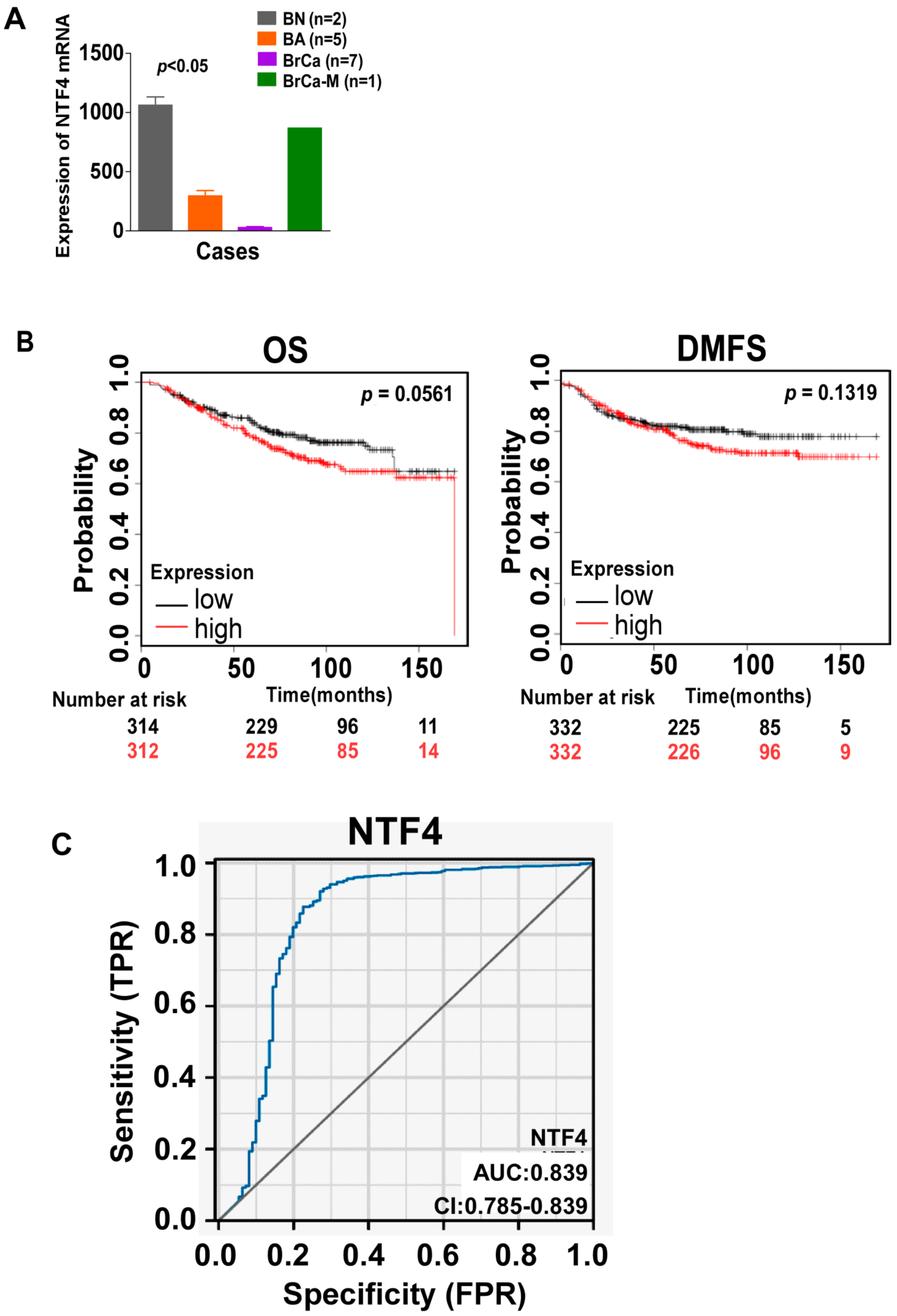
A
BN (n=2)
BA (n=5)
BrCa (n=7)
BrCa-M (n=1)
p<0.05
Expression of NTF4 mRNA
1500
1000
500
0
Cases
B
OS
p = 0.0561
Probability
0.0 0.2 0.4 0.6 0.8 1.0
Expression
low
high
0 50 100 150
Time(months)
Number at risk
314 229 96 11
312 225 85 14
DMFS
p = 0.1319
Probability
0.0 0.2 0.4 0.6 0.8 1.0
Expression
low
high
0 50 100 150
Time(months)
Number at risk
332 225 85 5
332 226 96 9
C
NTF4
1.0 0.8 0.6 0.4 0.2 0.0
Sensitivity (TPR)
NTF4
AUC:0.839
CI:0.785-0.839
0.0 0.2 0.4 0.6 0.8 1.0
Specificity (FPR)

Figure S2

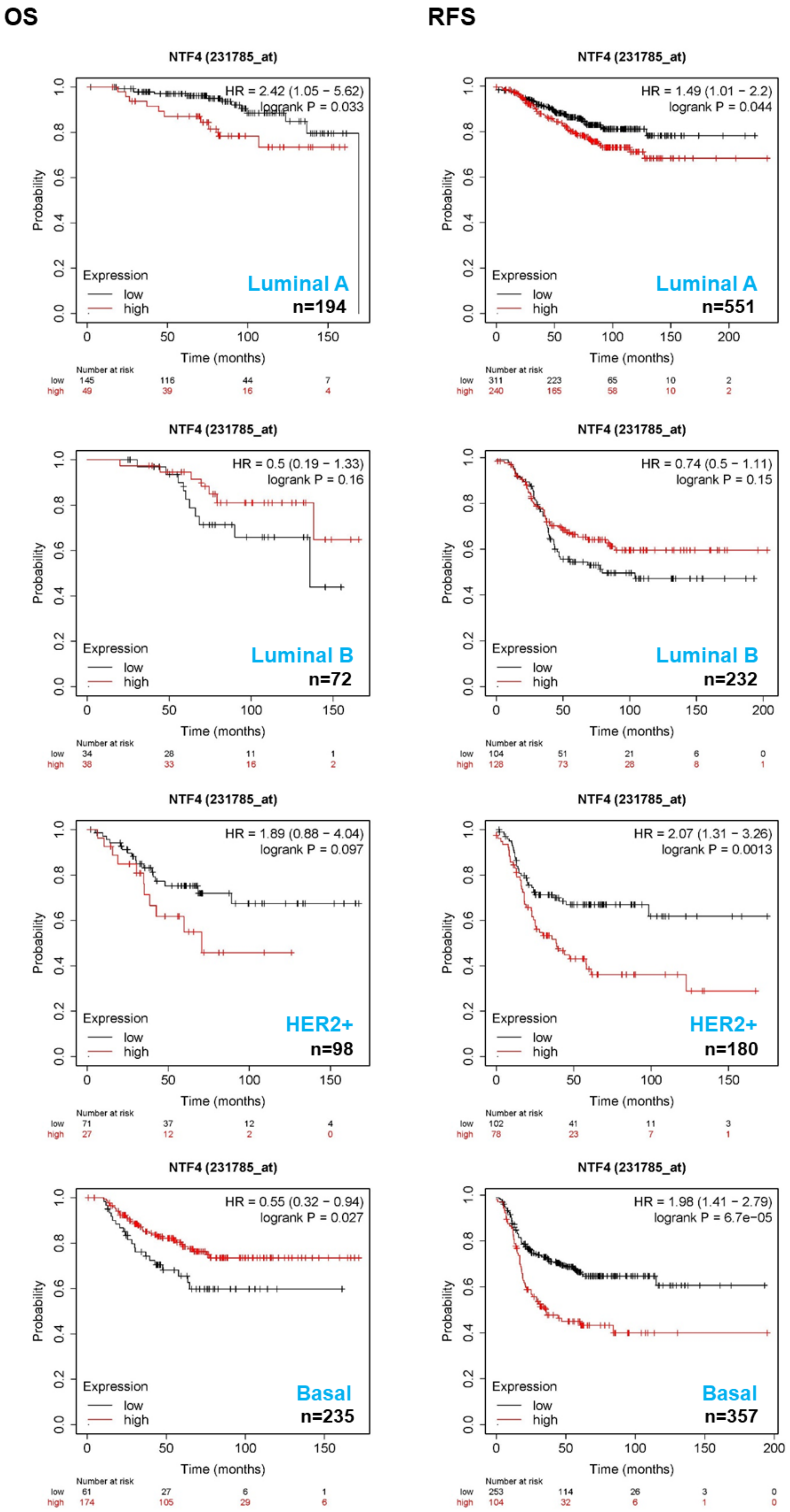

OS
RFS
NTF4 (231785_at)
HR = 2.42 (1.05 − 5.62)
logrank P = 0.033
Luminal A
n=194
HR = 1.49 (1.01 − 2.2)
logrank P = 0.044
Luminal A
n=551
HR = 0.5 (0.19 − 1.33)
logrank P = 0.16
Luminal B
n=72
HR = 0.74 (0.5 − 1.11)
logrank P = 0.15
Luminal B
n=232
HR = 1.89 (0.88 − 4.04)
logrank P = 0.097
HER2+
n=98
HR = 2.07 (1.31 − 3.26)
logrank P = 0.0013
HER2+
n=180
HR = 0.55 (0.32 − 0.94)
logrank P = 0.027
Basal
n=235
HR = 1.98 (1.41 − 2.79)
logrank P = 6.7e−05
Basal
n=357
Expression
low
high
Probability
Time (months)
Number at risk

Figure S3

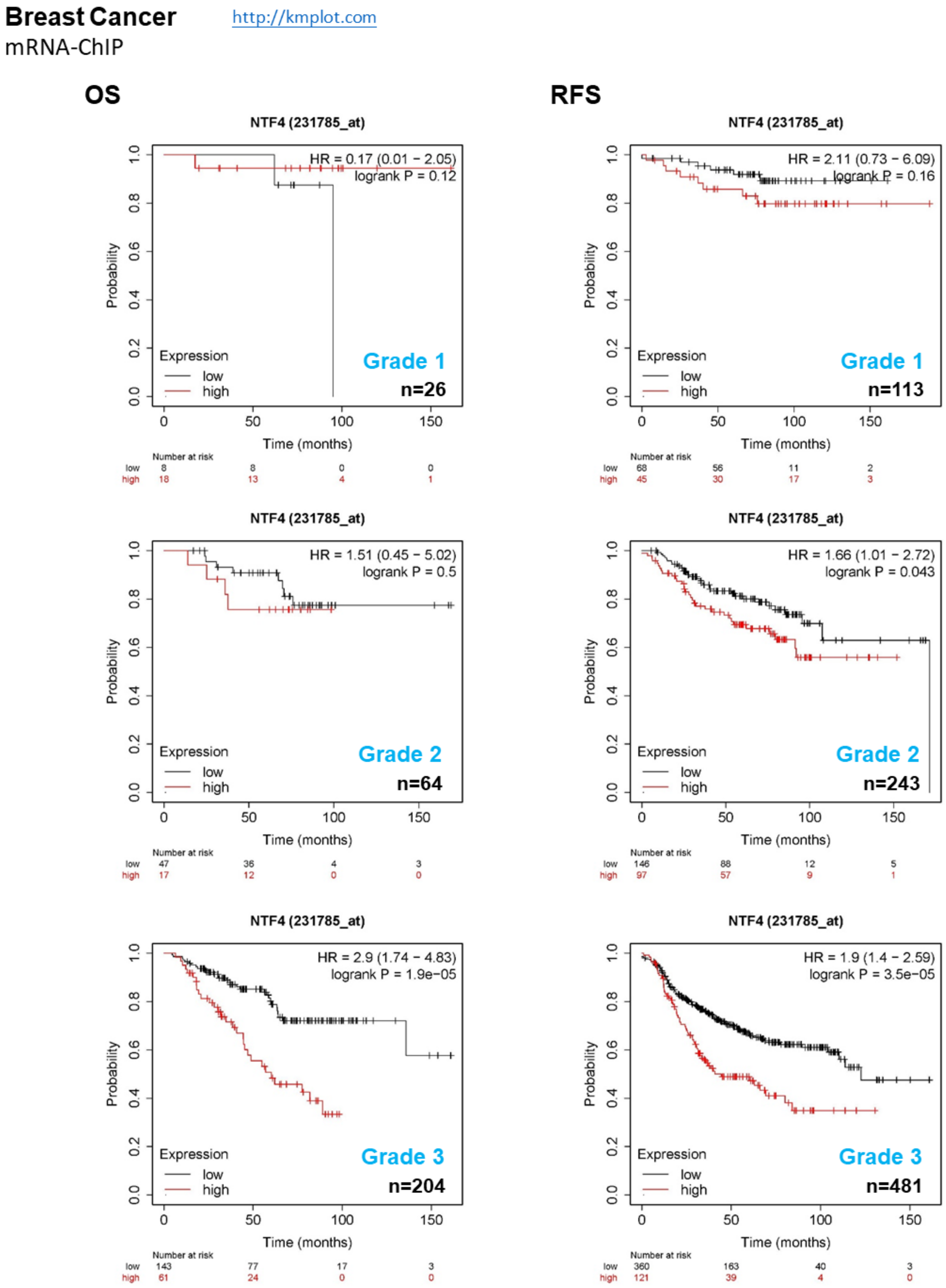

Breast Cancer
http://kmplot.com
mRNA-ChIP
OS
RFS
NTF4 (231785_at)
HR = 0.17 (0.01 − 2.05)
logrank P = 0.12
Grade 1
n=26
HR = 2.11 (0.73 − 6.09)
logrank P = 0.16
Grade 1
n=113
HR = 1.51 (0.45 − 5.02)
logrank P = 0.5
Grade 2
n=64
HR = 1.66 (1.01 − 2.72)
logrank P = 0.043
Grade 2
n=243
HR = 2.9 (1.74 − 4.83)
logrank P = 1.9e−05
Grade 3
n=204
HR = 1.9 (1.4 − 2.59)
logrank P = 3.5e−05
Grade 3
n=481
Probability
Time (months)
Expression
low
high
Number at risk

Figure S4

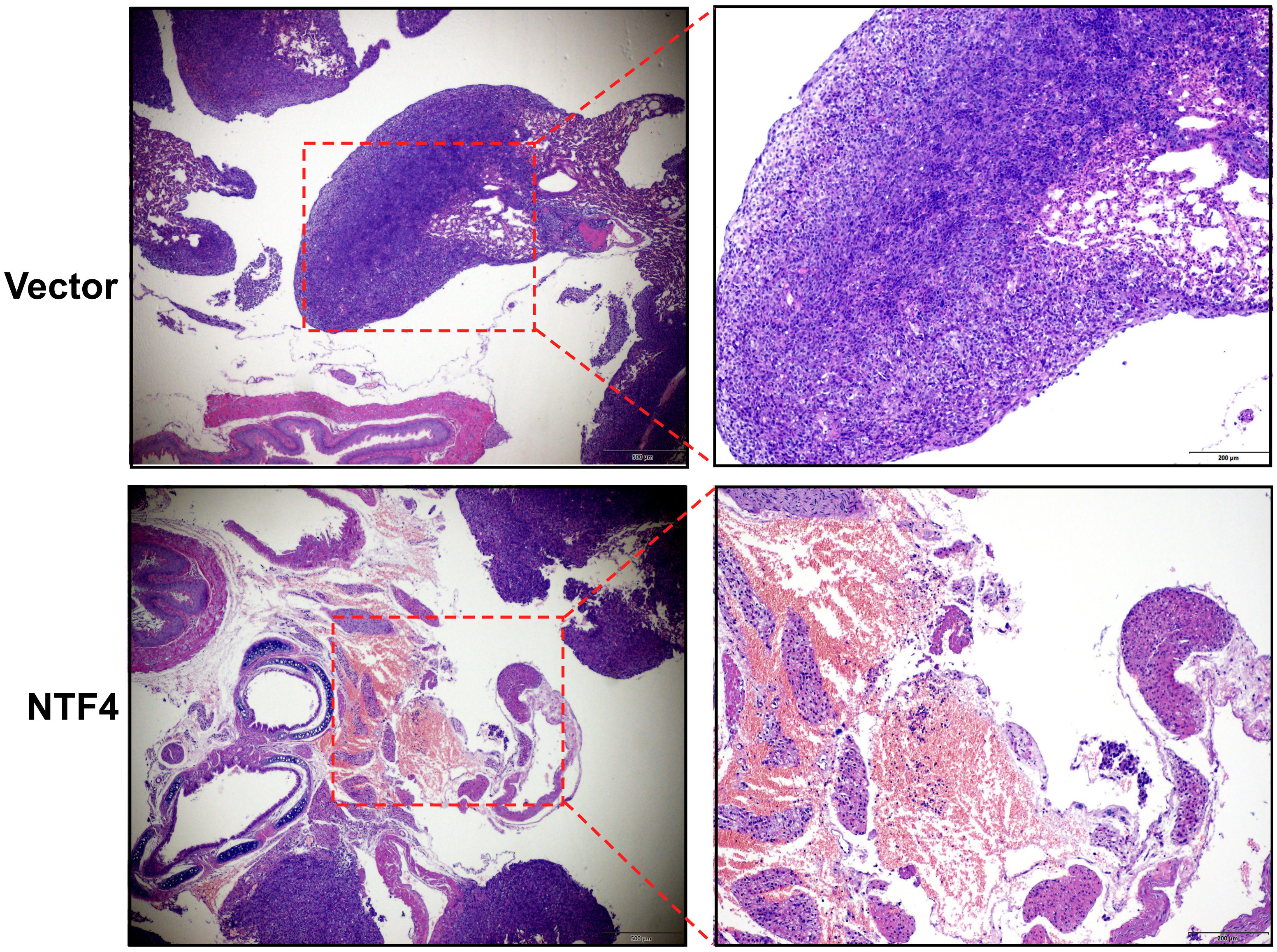

Figure S5

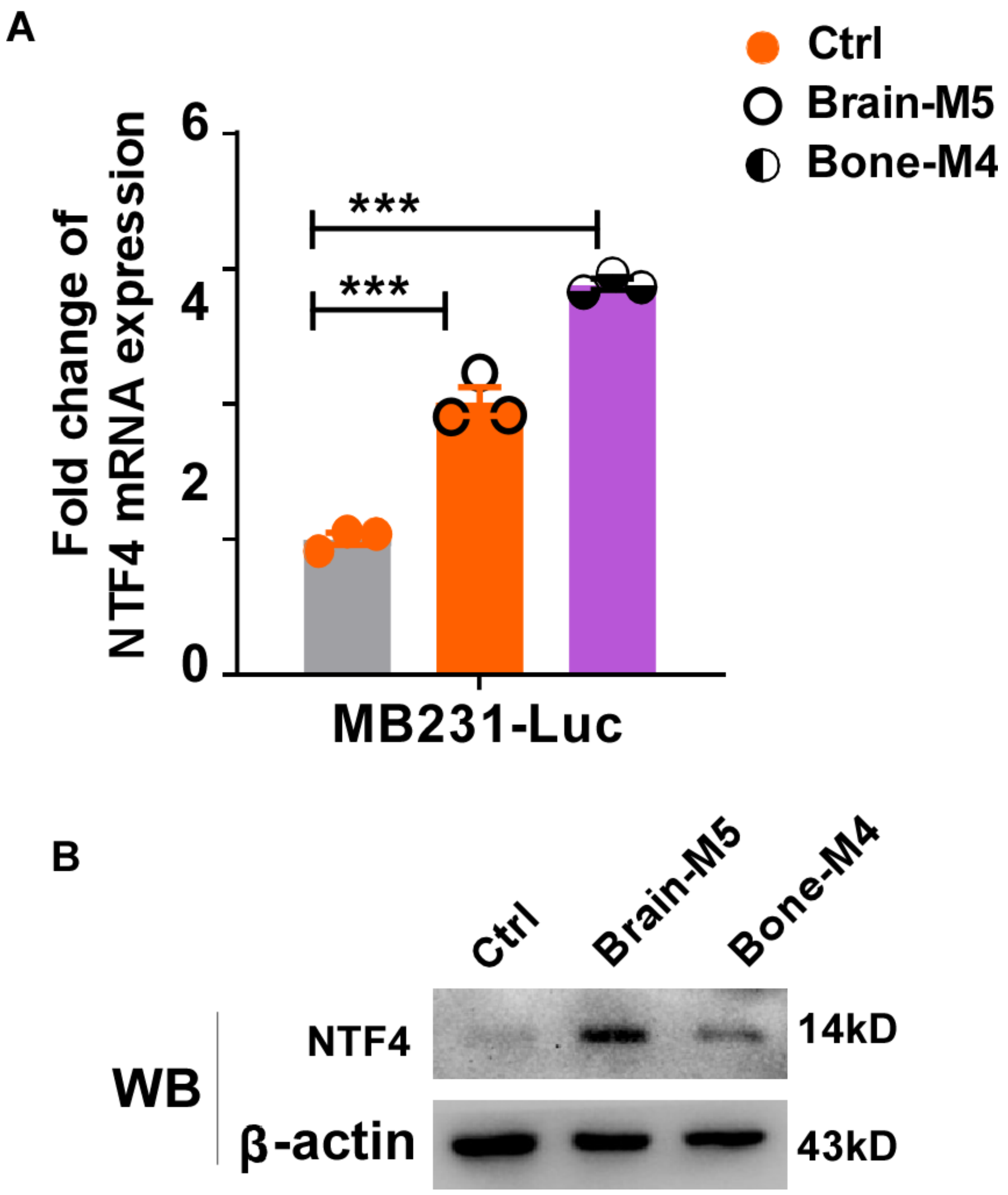
A
Ctrl
Brain-M5
Bone-M4
Fold change of NTF4 mRNA expression
6
4
2
0
***
***
MB231-Luc
B
Ctrl
Brain-M5
Bone-M4
WB
NTF4
β-actin
14kD
43kD

Figure S6

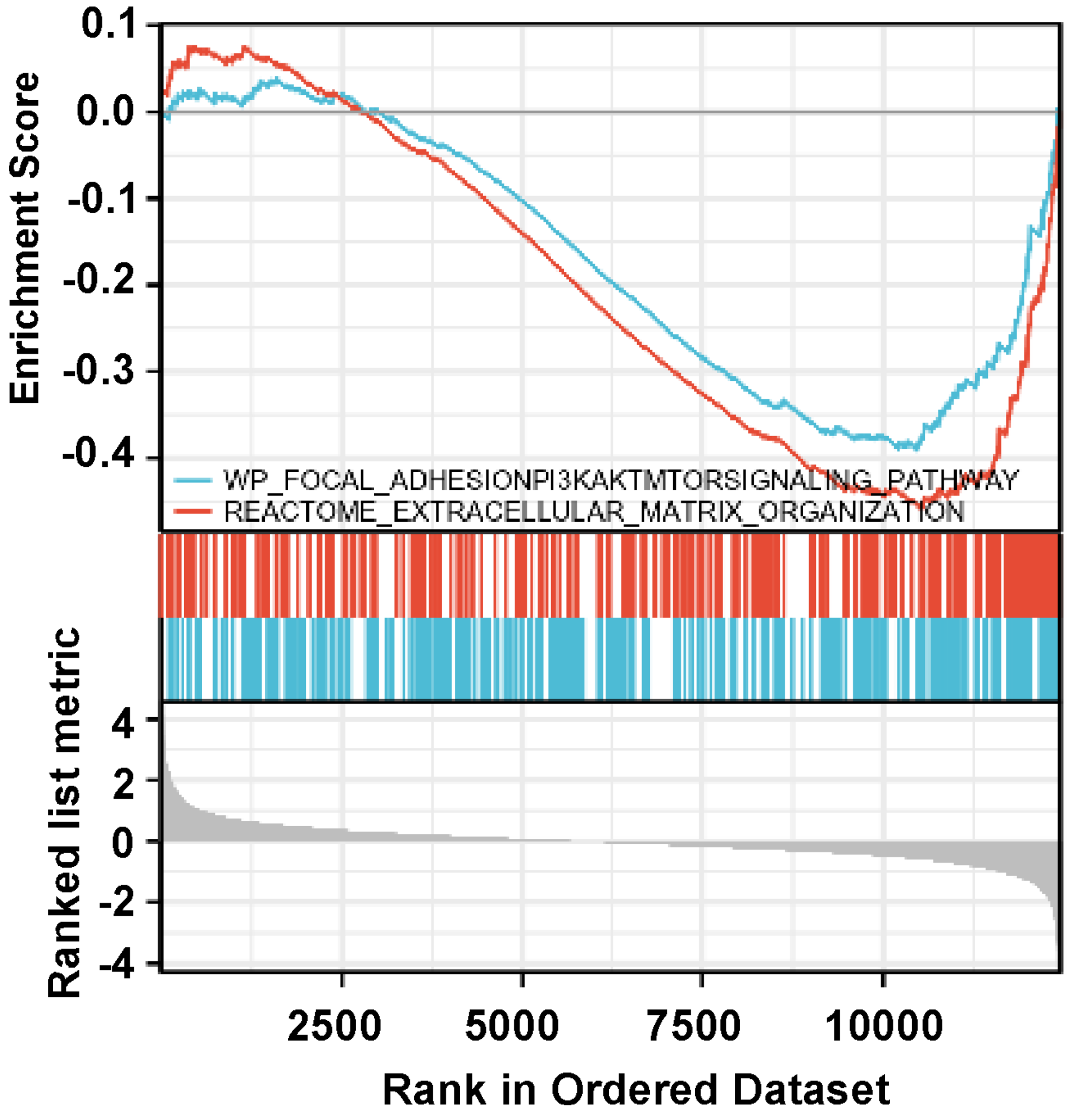
Enrichment Score
0.1
0.0
-0.1
-0.2
-0.3
-0.4
WP_FOCAL_ADHESIONPI3KAKTMTORSIGNALING_PATHWAY
REACTOME_EXTRACELLULAR_MATRIX_ORGANIZATION
Ranked list metric
4
2
0
-2
-4
2500
5000
7500
10000
Rank in Ordered Dataset

Figure S7

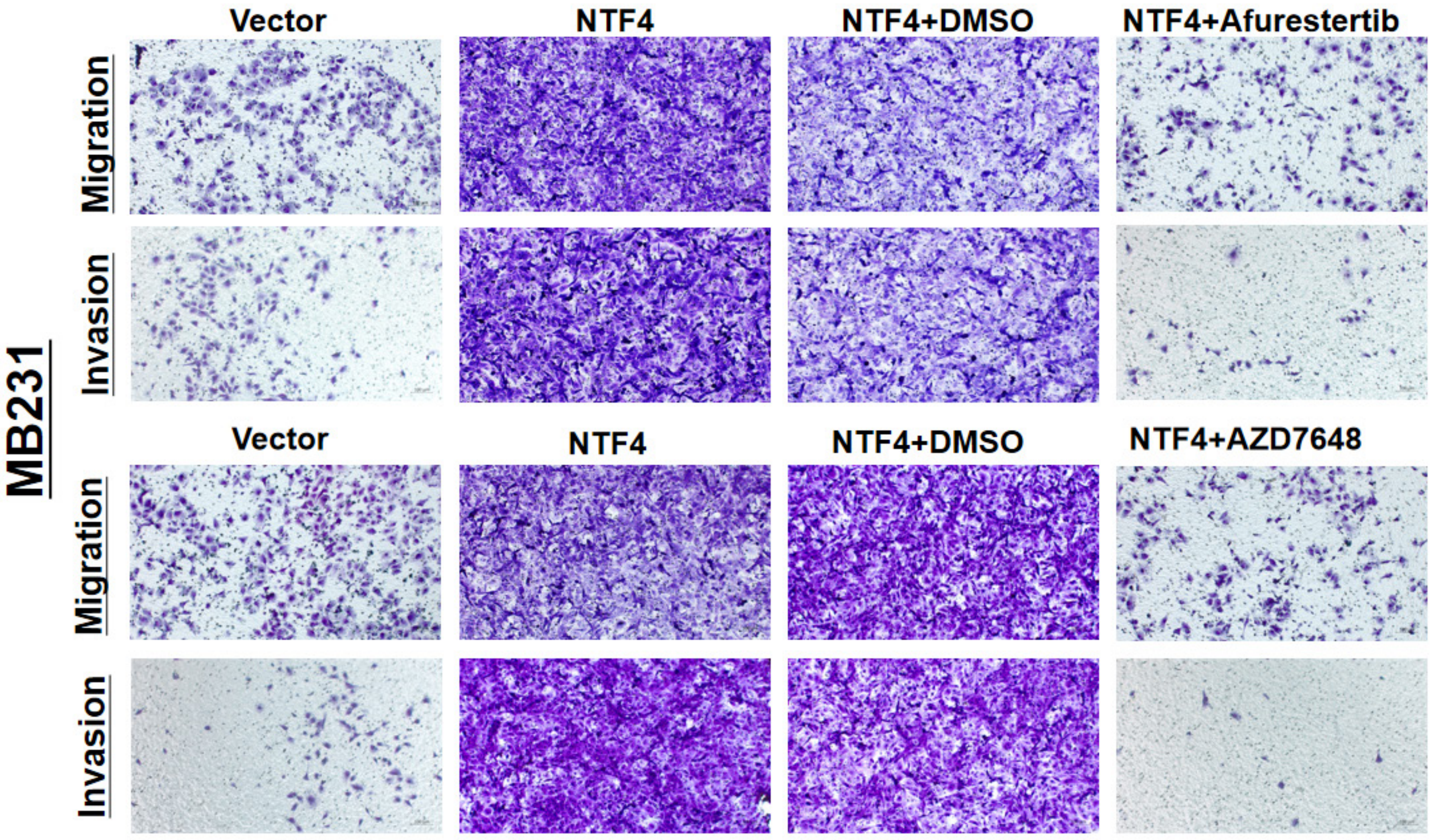

MB231
Vector
NTF4
NTF4+DMSO
NTF4+Afurestertib
Migration
Invasion
Vector
NTF4
NTF4+DMSO
NTF4+AZD7648
Migration
Invasion

Figure S8

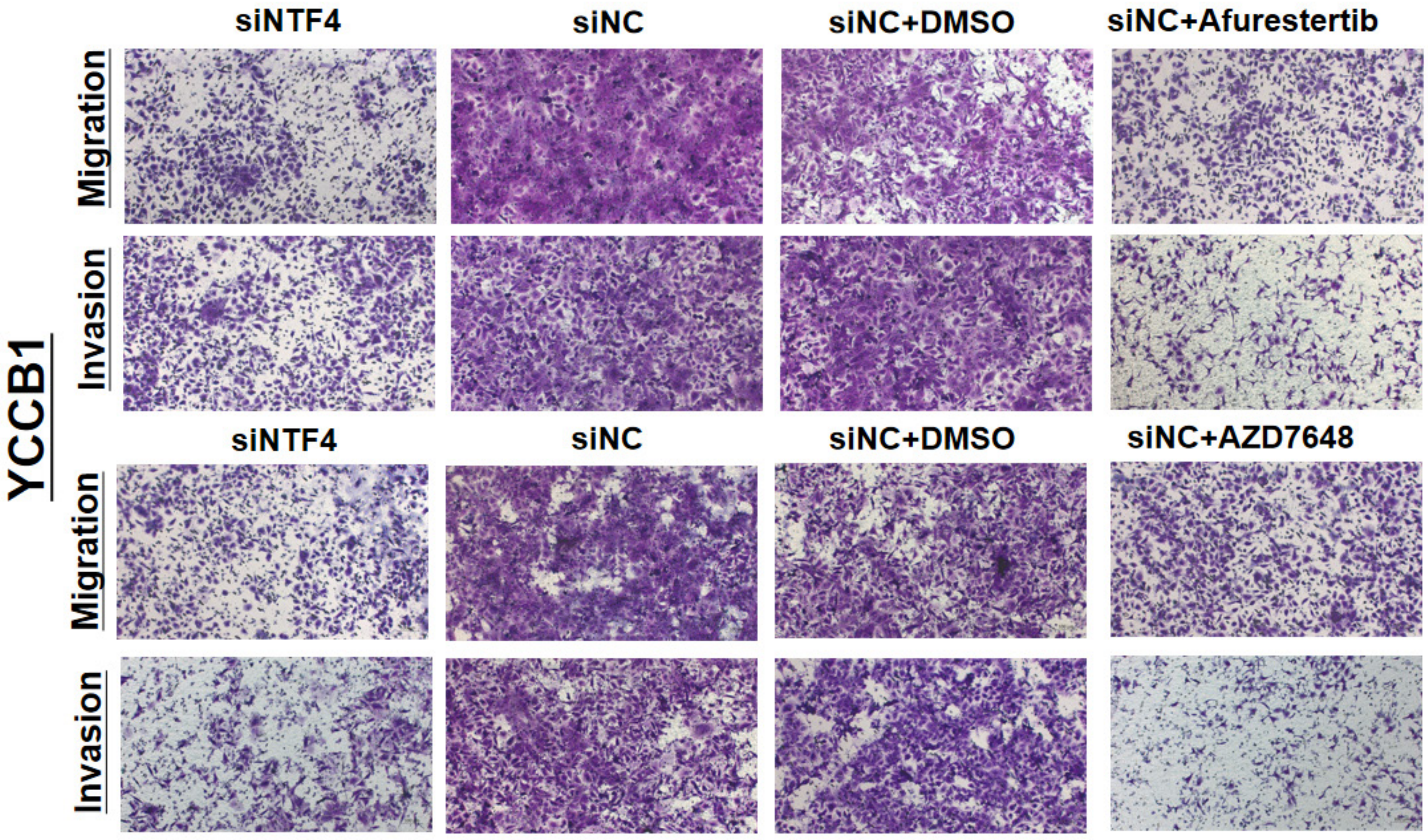
siNTF4
siNC
siNC+DMSO
siNC+Afurestertib
Migration
Invasion
YCCB1
siNTF4
siNC
siNC+DMSO
siNC+AZD7648
Migration
Invasion

Figure S9

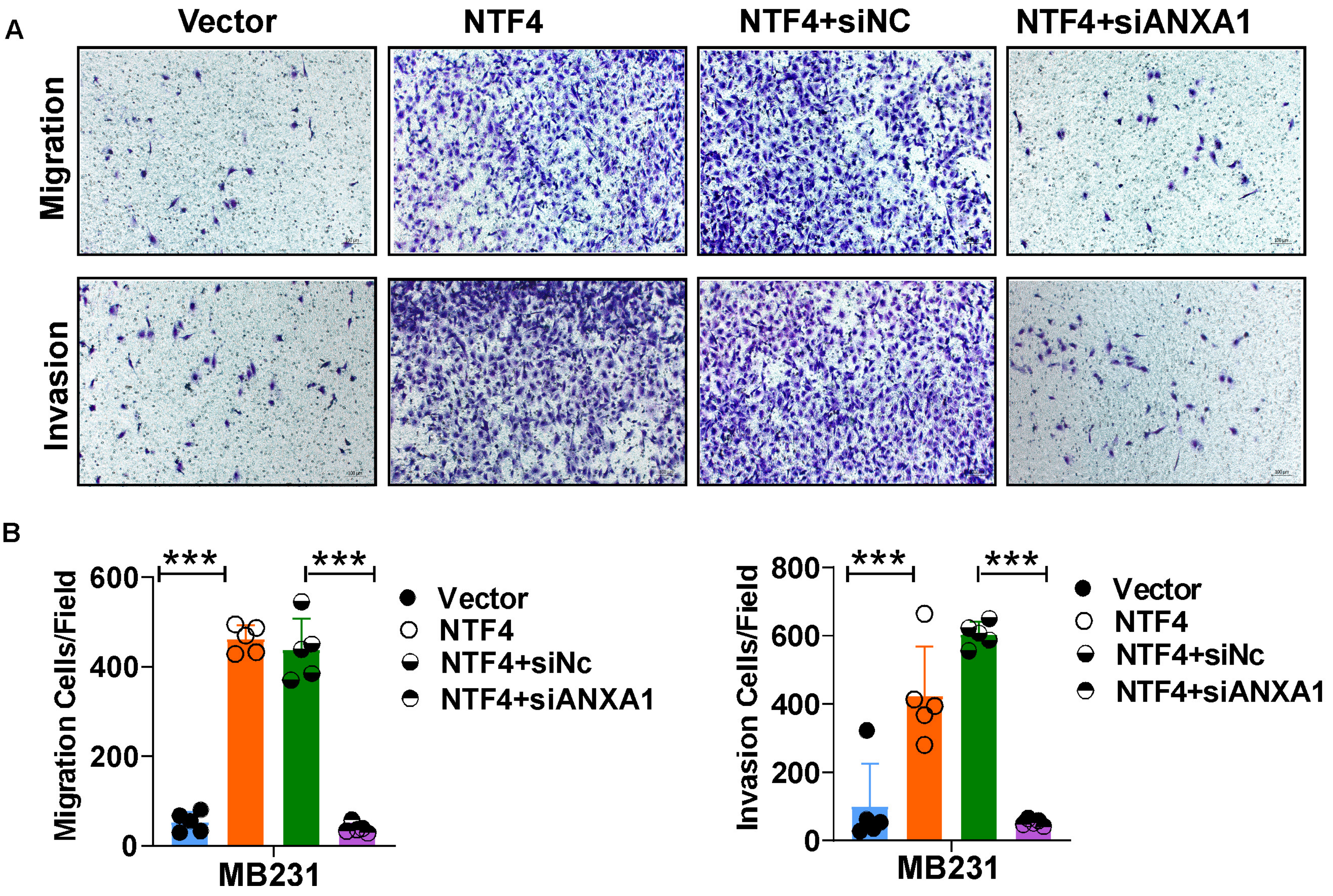
A
Vector
NTF4
NTF4+siNC
NTF4+siANXA1
Migration
Invasion
B
Migration Cells/Field
600
400
200
0
***
***
Vector
NTF4
NTF4+siNc
NTF4+siANXA1
MB231
Invasion Cells/Field
800
600
400
200
0
***
***
Vector
NTF4
NTF4+siNc
NTF4+siANXA1
MB231
